# Highlights on the Effects of Graphene-Family Materials Dispersion on Hydration and Mechanical Properties of Cement-Based Materials

**DOI:** 10.1021/acsomega.5c07530

**Published:** 2025-11-12

**Authors:** Isis Nayra Rolemberg Prudente, Hericles Campos dos Santos, Jander Lopes Fonseca, Jéssica Fernanda Ribeiro Oliveira, André Balbino Shibutani, Iara de Fátima Gimenez, Euler Araujo dos Santos, Ledjane Silva Barreto

**Affiliations:** † Graduate Program in Science and Engineering Materials, 74391 Federal University of Sergipe, São Cristóvão, Sergipe 49100, Brazil; ‡ Department of Sciences and Materials Engineering, Federal University of Sergipe, São Cristóvão, Sergipe 49100, Brazil; § Graduate Program in Chemical Engineering, Federal University of Sergipe, São Cristóvão, Sergipe 49100, Brazil; ∥ Graduate Program in Civil Engineering, Federal University of Sergipe, São Cristóvão, Sergipe 49100, Brazil; ⊥ Graduate Program in Electrical Engineering, 42511 Centro Universitário FEI, São Bernardo do Campo, São Paulo 09850, Brazil

## Abstract

Graphene-family materials (GFMs) have gained notoriety in academic research due to their ability to improve the mechanical properties of cementitious matrices and provide new functionalities to conventional systems, such as self-healing properties. However, the use of GFMs faces challenges due to the difficulty of these materials dispersing properly in cementitious matrices, often resulting in the agglomeration of nanomaterials. This tendency to agglomerate directly impacts critical properties of cementitious matrices such as workability and mechanical strength. Therefore, several research studies are being conducted to address dispersion issues and understand the interactions between GFMs and their matrices. However, the literature still lacks consensus on important topics, such as the parameter definitions that influence the cement hydration reaction rate, the factors affecting workability, the cement/GFMs interaction models, the type and optimal amount of GFMs to be used for each system, the characteristics of those GFMs (type and amount of functional groups, presence or absence of defects), and how differences in matrices and dispersants affect mechanical strength parameters. Finally, this work synthesizes the current knowledge on the effects of GFMs and their dispersion on cementitious properties, while also providing an assessment of the limitations of meta-analytical approaches in this field.

## Graphical abstract

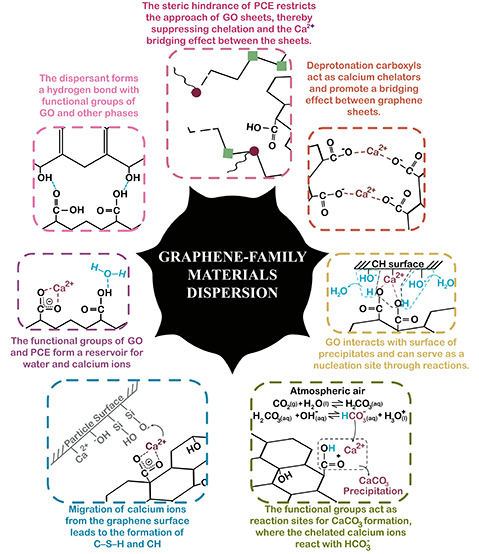

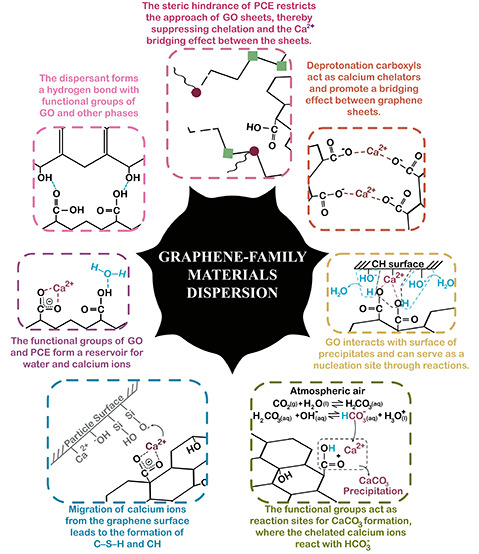

## 1 Introduction

The construction industry is an old and complex industry, operating through numerous systems that, despite being distinct, are interconnected, forming an intricate system that encompasses structural, electrical, hydraulic, sealing, and finishing systems. Just like the industry itself, the materials that constitute it are equally complex, such as cementitious matrices, which are essential and widely used in almost all construction systems. Although widely used, these materials are expected to perform increasingly better to become more efficient,^1–3^ eco-friendly,^4–6^ and multifunctional.^7–15^

One way to ensure these attributes is through the use of additives. Among these additives, graphene-family materials (GFMs) stand out, including graphene nanoplatelets (GNPs), graphene oxide (GO), and reduced graphene oxide (rGO). Despite the great promise of these nanoadditives to improve cementitious matrices, some problems hinder their full utilization, such as dispersion difficulties.^16–21^ In alkaline matrices, the high concentration of alkaline ions, such as calcium ions, can interact with the functional groups of GFMs, leading to sheets agglomeration.^22,23^ Additionally, the high pH of the medium can chemically reduce GFMs, such as GO, by removing oxygenated functional groups. Once partially reduced, the predominant interactions between the remaining functional groups are *van der Waals* interactions, leading to the agglomeration of nearby GFMs.^22,24^

One strategy to overcome these problems is the incorporation of dispersants,^24–26^ among which the more often discussed in the literature are polycarboxylate-based superplasticizers (PCEs),^18,26–29^ sodium dodecyl benzenesulfonate (SDBS),^27,28^ naphthalene-based superplasticizers (NSs),^2,25,29^ and active silica.^26,30,31^ Another alternative is covalent functionalization,^32–35^ which can occur in two ways. The first involves the modification of GFMs through reactions with an agent that introduces functional groups into the GFMs (such as triethanolamine), which reacts with the epoxy groups (−C–O–C−) of the basal planes, adding extra hydroxyl (−OH) groups to the sheet edges. In addition, GFMs can be modified using nanosilica or siloxanes, which react with hydroxyl and/or carboxyl (−COOH) groups, forming bulky groups capable of promoting steric hindrance between the GFM layers.^32,34,36^ The second approach consists of the functionalization of the system components themselves (aggregate, reinforcement, or pozzolanic material), in order to establish a connection between the component and the GFMs through the reaction of amine groups with the hydroxyl groups available on both surfaces.^37–41^

Therefore, the introduction of nanoparticles such as GFMs adds further complexity to an already complex system like cement. In this context, there are the effects of nanoparticles on the properties of cement (microstructure, mechanical properties, durability) and the interactions with cement phases (which describe the hydration mechanisms).^29,42,43^ Both are sources of divergences in the literature about the effect of GFMs on the cementitious matrix. The understanding of the incorporation of these nanomaterials is also limited by the variability of the system (paste, mortar, and concrete) and by the availability of information about the GFMs characteristics as well as their dispersion methodology.

For example, Raman spectroscopy analyses have revealed an increase in structural defects and dangling bonds in some dispersion methods, particularly in the ultrasound method process,^44,45^ depending on the duration and the applied power. This type of method increases the degree of disorder of the sheets while simultaneously promoting the fragmentation of GNPs, preferably along regions with vacancies or edge defects, due to the action of shock waves and shear forces generated by ultrasonic cavitation.

While previous reviews have established the fundamental potential of GFMs to enhance cementitious composites, such as mechanical properties and durability,^46–48^ they often present generalized conclusions. Building on this premise, the present review seeks to advance the discussion by pursuing three specific objectives: (i) Review the effects of GFMs on hydration mechanisms, microstructural evolution, and durability parameters. (ii) Identify and contextualize optimal GFMs dosages for different matrices. (iii) Analyze the correlation between dispersion methodologies (focusing on dispersant type and dosage) and the achieved mechanical performance. Finally, this work provides an overview while simultaneously offering a critical perspective on the challenges of meta-analysis in this field, pointing out limitations on data compilation and highlighting the necessity of standardizing methodologies.

## 2 Statistical Analysis Methodology

All of the statistical analyses were implemented in Python Programming Language using the statsmodels package for ANOVA (Analysis of Variance) and LOESS (Locally Estimated Scatterplot Smoothing), and the piecewise_regression package for piecewise regression.

For the analysis presented in Figure 2 (obtained exclusively from Supporting Information Table S.1), the optimal graphene/dispersant (G/D) ratio was obtained by applying LOESS (Analysis of Variance) and piecewise regression.^49,50^ The former is a nonparametric method that gives weights scores to points in an *n*-dimensional space according to its proximity to the data analyzed.^50^ In the case of this work, this statistical regression method creates curves considering the larger weights scores attributed to the model, fitting the tendency of the study phenomena.^49^ The python LOESS regression function receives as the main inputs the data from the *x*- and *y*-axes. From these data, each point of an *n*-dimensional space is evaluated by a score based on the lower distance between the space points and the data points.

The second statistical tool applied to the data was the piecewise regression, which divides the *x*-axis into different segments according to the data profile, a region fitted by a mathematical equation (polynomial, linear curves, etc.) that follows the data locally, generating data clusters.^51^ The points of intersection between those segments or functions are called knots, where there is a change in curve tendency and, consequently, in the polynomial curve to fit the data flow. Thus, the method creates many local mathematical equations that are limited by breaking points or knots. Also, increasing the number of break points did not alter the overall trend. The piecewise regression function in Python takes the data for the *x*-axis and *y*-axis as well as the number of break points (or knots) by which the *x*-axis should be divided. Based on that, the algorithm fits the data using different polynomial equations according to its tendency, where the limit of each polynomial is the specified knot. It is important to point out that the first method predicts exclusively on the basis of data points, while the second method is based on linear functions to fit the data.

To the ANOVA function, the data of different columns were taken in pairs, resulting in the variables’ *p*-values being analyzed in this work. The unique limitation posed by ANOVA is that the dispersant and visual analysis data, as categorical variables (represent type or quality of data instead of numerical quantities) cannot be used as an output variable but only by input data for the model. In Figure 17 (obtained from Table S.2), the data were distinguished according to the type of dispersant employed in each study. For this analysis, different types of cementitious matrices were grouped together without a distinction. Only the material combinations that resulted in the best mechanical performance were considered. The data from this table were also used in the statistical analysis in which the correlation between the presented factors and compressive strength was evaluated.

## 3 Limitations on Data Compilation for Determining the Optimal Dispersion Methodology

The literature presents several methods to disperse GFMs, as noted in the introduction chapter.^2,25,29^ These methods influence the effects of GFMs on the properties of modified cementitious matrices. Such effects are observed in the microstructure, workability, mechanical strength, and durability of these matrices. As a basis for the discussion of these topics in subsequent chapters, this section addresses dispersion methodologies and investigates the limitations associated with compiling heterogeneous data to determine optimal graphene-dispersant ratios. This study focuses specifically on GO.

Table 1 presents a compilation of the dispersion parameters for GO reported in the literature, including the types of dispersants, the GO/dispersant (G/D) ratios, the dispersion methodologies employed, and the techniques used to characterize dispersion efficiency (UV–vis, ζ potential, and visual analysis). For subsequent quantitative analyses (Figure 1 and statistical analysis), it was used exclusively by data obtained by UV–vis spectroscopy or by binary visual analysis (dispersed/agglomerated).

**Table 1:** Dispersion Methodologies and Related Efficiency Parameters for the GFMs^a^ ^,^ ^b^

Dispersant type	GFM amount	G/D	Methodology for dispersion	UV–vis (Abs)	ζ potential (mV)	Visual anal	Observation	ref
–	2.5 mg	1:0	–	0.405	–1.1	–	PCE was used as the main dispersant and MO as an auxiliary. UV–vis data were obtained within 20 min. There was no information about the pH during the ζ potential experiment.	^52^
MO and PCE	2.5 mg	1:0.5	GO and MO were stirred for 5 min at 3000 rpm, then mixed with PCE in 125 mL of saturated Ca(OH)_2_ solution.	0.400	–2.22	–		
		1:1		0.440	–2.75	–		
		1:1.5		0.425	–2.65	–		
		1:2		0.404	–2.15	–		
		1:2.5		0.385	–2.01	–		
								
	0.05 g	1:0		0.900			It was not clear how long after dispersion the UV–vis analysis was performed.	^53^
Sika-PCE	0.05 g	1:0.5	GO was mixed with water and the superplasticizer and magnetically stirred at 400 rpm for 15 min and, then, sonicated at 650 W for 30 min. The experiment was conducted in water.	0.920				
		1:1		1.015				
		1:2		0.960				
		1:4		0.905				
APEO	0.05 g	1:1		1.016		A	It was not clear how long after dispersion the UV–vis and visual analysis were performed.	
TNWDIS				1.065		A		
I-PCE				0.834		D		
II-PCE				0.556		A		
Silane-PCE				0.943		D		
Sika-PCE				1.002		D		
								
GOS	–	–	Nanosilica was deposited on GO by in situ hydrolysis and condensation of TEOS in water.		–56.7		The nanosilica-decorated graphene showed negligible change in ζ potential yet demonstrated superior visual stability in saturated Ca(OH)_2_ solution, unlike pure graphene.	^33^
	0.04 mg/mL				–52.2			
GOS	–	–	Nanosilica was deposited on GO by in situ hydrolysis and condensation of TEOS in a saturated Ca(OH)_2_ solution.			A		
	0.04 mg/mL					D		
								
		1:0			–185			^54^
SL	1 mg/mL	1:5	GO was mixed with the dispersant using electromagnetic stirring, followed by a 30 min ultrasonic bath in cement pore solution.			A	The ζ potential values presented are for pH around 13.	
		1:10			–160	A		
		1.20				A		
PNS		1:5				A		
		1:10			–90	A		
		1.20				A		
PCE		1:5				A		
		1:10			–55	D		
		1.20				D		
PCE	1mg/mL	1:1	GO was mixed with the dispersant using electromagnetic stirring, followed by a 30 min ultrasonic bath in water.		–27.5		The ζ potential values presented are for pH around 13.	
		1:2			–24.0			
		1:3			–22.5			
		1:4			–18.0			
		1:5			–16.2			
								
PCE	0.003 g	1:0	The GNPs, in paste form, were mixed with water and PCE and sonicated for 60 min. The dispersion experiment was conducted in water.	2.48				^55^
		1:22		2.53				
		1:55		2.56				
		1:77		2.6				
		1:99		2.63				
		1:132		2.63				
		1:176		2.68				
		1:0		–		A	The visual analyses are from 24 h dispersions. The time used to sonicate the dispersions for the UV–vis and visual analysis assays was not clear.	^56^
PCE-2		1:1	GO and PCE were mixed, electromagnetically stirred for 30 min, and, then, sonicated. The dispersion experiment was conducted in cement pore solution.	0.3		A		
		1:3		2.5		D		
		1:5		2.3		A		
PCE-4		1:1		2.75		A		
		1:3		3.2		D		
		1:5		2.6		A		
PCE-6		1:1		1.8		A		
		1:3		3.6		D		
		1:5		3.3		D		
PCE-44		1:1		1.4		A		
		1:3		3.0		D		
		1:5		2.8		D		
								
SGO	0.04 mg/mL	–	GO, water, and ethanol were mixed for 30 min, followed by the addition of APTS and stirring for 24 h in a saturated Ca(OH)_2_ solution.	0.23		A	The UV–vis analysis is from 120 min while the visual analysis is from the initial time.	^32^
		–		0.9		D		
EVA	0.1 mg/mL	1:0	EVA was dispersed in water, GO was added, and the mixture was ultrasonicated for 10 min in cement pore solution.	0.18		A	The UV–vis analysis is from 30 min while the visual analysis is from the 60 min.	^57^
		1:100		0.52		D		

a Obs_1_: A, agglomerated; D, dispersed; G/D = GFMs/dispersion ratio; MO = Methyl orange; GOS = GO functionalized with nanosilica; SL = sodium lignosulfonate; PNS = polycondensate of β-naphthalenesulfonate; SGO = GO functionalized with silane; EVA = ethylene-vinyl acetate.

b Obs_2_: This analysis was performed only with GO.

**Figure 1:**
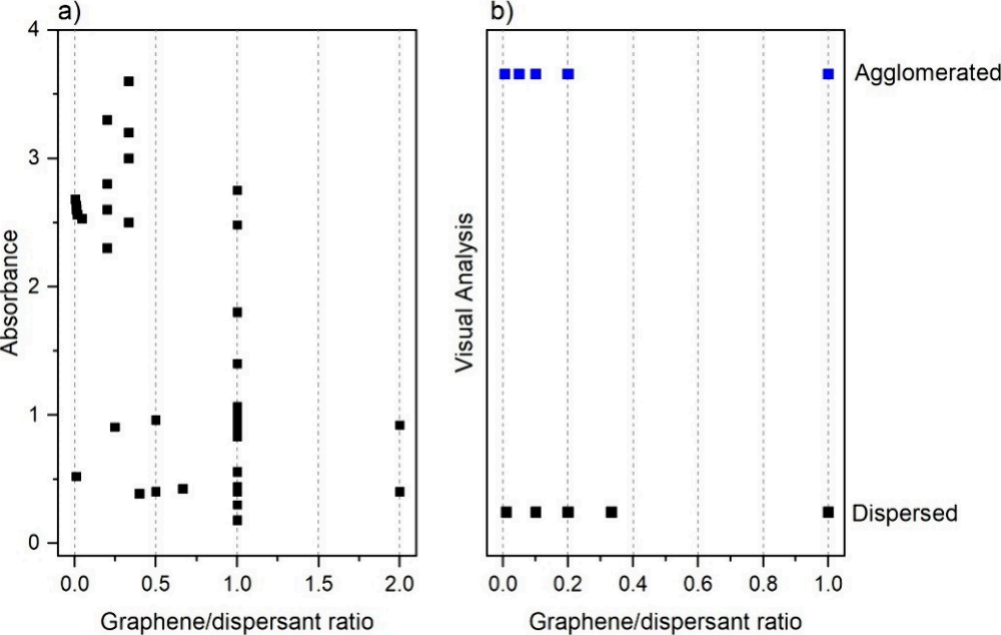
Correlation between the G/D ratio and dispersion efficiency. (a) Absorbance (λ = 220–230 nm) as a function of the G/D ratio, measured by UV–vis. (b) Result of the binary visual analysis (dispersed/agglomerated) as a function of the G/D ratio.

The ζ potential data were excluded from the statistical analysis due to the significant heterogeneity in the experimental conditions reported across different studies (e.g., divergent and often unreported pH values), which prevents a direct and reliable comparison.

Observation of the data in Figure 1a, which relates absorbance (a response parameter for dispersed GO concentration) to the G/D ratio, suggests that the most effective proportions for GO dispersion lie within the range of 0.25–1 (equivalent to a 1:4 to 1:1 dispersant:GO ratio). This can also be corroborated by an analysis of variance (ANOVA), which returned a *p*-value of 0.000013 for the “G/D ratio” factor. This highly significant result (*p* ≪ 0.05) allows for the rejection of the null hypothesis, indicating that the GO/dispersant ratio exerts a statistically significant influence on the material’s dispersibility when assessed by UV–vis.

Although excluded from quantitative analysis due to lack of standardization, the ζ potential data presented in Table 1 show a general trend qualitatively. More negative values (generally <−30 mV) or more positive values (>+30 mV), often associated with stable dispersions, were observed within the G/D ratio range of 1:1. However, the results obtained by quantitative and statistical analysis for UV–vis data are corroborated, Figure 1.

In turn, Figure 1b correlates the same G/D ratio with dispersion stability results obtained by binary visual analysis over time. Visually, it is not possible to discern a clear trend among the variables. This observation is confirmed statistically by a second ANOVA, which resulted in a nonsignificant *p*-value of 0.454. This indicates that, within the scope of this methodology, the G/D ratio is not identified as a determining factor for the “dispersed” or “agglomerated” outcome.

The discrepancy between the results from the two techniques is notable and suggests that binary visual analysis for statistical treatment, in its current form, is an insufficient tool for capturing the influence of subtle dosage parameters. This limitation can be attributed to (1) loss of information—the binary nature (yes/no) disregards gradations of dispersion/agglomeration; (2) subjectivity—the classification is subject to observer interpretation, without standardized or quantitative criteria; (3) low sensitivity—the human eye is incapable of detecting subtle differences in the concentration of dispersed GFMs that are perfectly quantifiable by instrumental techniques such as UV–vis. The visual evaluation of color and turbidity is subjective, varying with the human eye’s perceptual limits (380–780 nm)^58^ and an observer’s acuity. This subjectivity results in uncontrollable measurement errors that complicate cross-study comparisons. For future studies, it is recommended to adapt a classification scale that contemplates different degrees of dispersion and agglomeration (e.g., from 1 to 5), accompanied by a protocol with standard reference images for each level of the scale, thereby mitigating subjectivity and increasing the analytical resolution of the method.

It is worth emphasizing that although the G/D ratio is an important factor, other factors need to be considered, such as the dispersion experimental procedure and the different types of dispersants. An analysis of this type could suggest the required chemical and structural nature of the dispersant for the effective dispersion of GFMs. More detailed analyses are necessary, even among dispersants of the same type such as PCEs. These are commercially available with a variety of characteristics (e.g., charge density, main chain length, and side chain size), which can significantly influence their effectiveness in dispersing GFMs. However, such parameters are often neglected or not detailed in the literature, hindering comparative analysis.

In this work, it was decided not to perform a formal statistical analysis to correlate the type of dispersant with dispersion efficiency, because the majority of the literature works use some type of PCE, while other classes of dispersants (APEO, EVA) are under-represented. A direct comparison, with such unbalanced sample sizes between dispersant classes, would be methodologically unfair and potentially misleading. Therefore, future, more robust investigations into the influence of the specific dispersant chemistry depend on the generation and compilation of a broader, balanced, and standardized data set.

However, considering that most of the data compiled in this work refer to the dispersant PCE, an attempt to identify the optimal graphene/dispersant (G/D) ratio for this compound was made by applying two different statistical analyses: the LOESS (locally estimated scatterplot smoothing) regression and the piecewise regression^49,50^ (Figure 2). The methodology used for both the analysis of variance (ANOVA) and the following statistical analyses is described in the Supporting Information.

**Figure 2:**
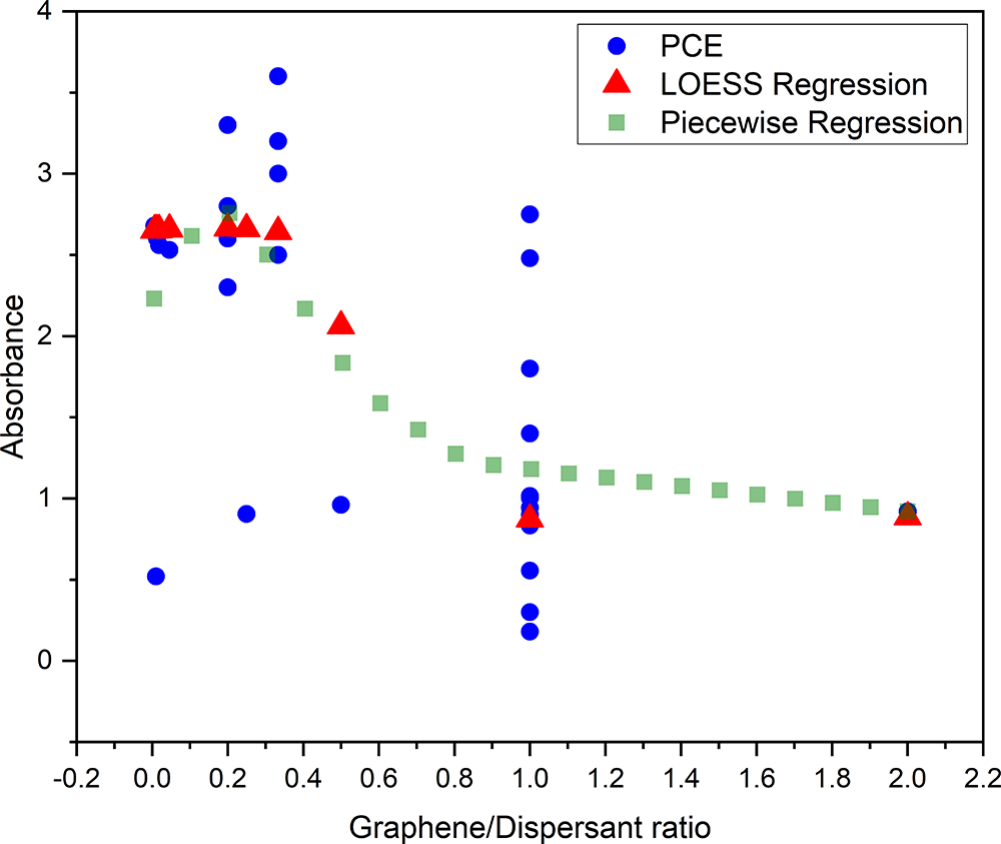
Correlation of the PCE with the graphene/dispersant ratio.

The first method predicts based exclusively by data points, while the second method is based on linear functions to fit the data. Thus, the study of this work analyzes the G/D ratio as a function of the absorbance according to the two methods to ensure better results and confirm the tendency between variables. In that context, the PCE as a function of G/D ratio is evaluated in Figure 2, where the knots of piecewise regression are in 1:3, 1:1, and 2:1. As can be observed, the decrease in G/D proportion leads to a better PCE result (0 < G/D < 1/3). On the other hand, the polynomial fit of the piecewise regression model results in saturation in PCE for G/D > 1:4. As a consequence, both models and the data itself point out a better G/D proportion for values lower than 1:3. Additionally, from the piecewise regression curve, a proportion between zero and 1:4 can optimize the absorbance.

However, it is important to recognize the inherent limitations of this approach, as the compiled data exhibit heterogeneous experimental conditions. These include variations in initial GFMs concentration, type of medium analyzed, PCE properties (e.g., molar mass and charge density), GFMs functional groups, and dispersion testing protocols. Therefore, this represents a nonstandardized experimental approach across different studies that also fails to account for the physicochemical aspects of colloidal systems. For example, the absorbance value, used as a reference for the concentration of the dispersed material, is dependent on the initial concentration of the dispersion. Consequently, higher absorbance values may just reflect a higher initial concentration of graphene and not necessarily a higher dispersion efficiency for a given G/D ratio.

Therefore, establishing a universally applicable G/D optimal ratio from such a diverse data set is questionable. A fairer and more reliable approach would involve comparing studies that share identical methodological conditions or would require a colossal volume of data meticulously categorized for every possible combination of variables. Therefore, the statistical trend observed here may reveal less about a universal optimal parameter and more about the dosage ranges most frequently investigated in the literature. This exercise demonstrates that the search for generalized correlations in nonstandardized data may generate more noise than insight, masking methodological particularities that are, in fact, determinants of the outcome.

This same principle extends and directly connects to the analyses presented in subsequent sections. The significant variations observed in the effects of GFMs addition on hydration, microstructure, workability, mechanical strength, and durability of cementitious matrices are also a direct reflection of the dispersion achieved in each specific experimental methodology. Thus, the search for universal cause–effect relationships remains a challenge, highlighting the need for methodological standardization and a clear and complete description of the methodological parameters executed in each research study.

## 4 Influence of GFMs on Cement Hydration

The complexity of cement hydration makes the investigations of the influence of the GFMs challenging as different matrices including pastes, mortars, and concretes behave differently in the presence of additives such as GFMs and superplasticizers. This diversity of behaviors reflects on the lack of consensus regarding changes in the hydration reaction, microstructure, mechanical properties, and interaction models between GFMs and such matrices. Thus, distinct outcomes of the hydration process of cement matrices in the presence of GFMs can be found, as presented in Table 2. For instance, GFMs are often depicted as hydration accelerators, particularly GO, while conflicting effects were described for GNP and low-functionalized GO.

**Table 2:** Comparative Table of the Effect of Different GFM and Dispersion Methodologies on the Hydration Rate of Cementitious Matrices^a^

Type of GFM	Dispersion method	Matrix	Effect on hydration	Ref
GNP with different thicknesses (2, 6–8, and 11–15 nm)	The material was dispersed via ultrasonication for 3 min in an aqueous solution containing 1.66% PCE-based superplasticizer. No data were provided on the power, frequency, or model of the ultrasonic processor.	Mortar of OPC	GNPs with greater thicknesses accelerated the hydration rate and those with smaller thicknesses delayed the acceleration.	^59^
GNP with thickness (thickness, <5 nm)	The materials were dry mixed with ordinary Portland cement for 72 h. Subsequently, water was added to the mixture and blended for 5 min using a high-shear mixer. No information was provided regarding the power or model of the mixer.	Mortar of OPC	Variation in GNPs amount did not affect hydration rate.	^60^
GNP (diameter < 2 μm; thickness, 1–2 nm)	GNPs were predispersed using various surfactants (PCE, SC, SDBS, F127) via sonication, with amplitudes ranging from 20 to 100% and durations from 15 to 120 min, at surfactant-to-GNP mass ratios of 0.5–2.5.	Paste of OPC	The use of SDBS or PF127 presented no significant improvement in hydration rate	^28^
GO with low oxygen content (thicknesss = 1 atomic layer; flake size = 0.5–5 μm)	The material was predispersed with PCE using an ultrasonic device (UZDN-2T, UkrRosPribor, Belgorod, Russia) for 2 min at a frequency of 44 kHz.	Paste of OPC	Slight delay in hydration rate	^61^
GO (lateral size = 100–1000 nm; thickness = 0.7 nm)	Dispersed with aid of PCE (0.5% of cement). No details were provided regarding the order of addition of the reagents, the dispersion method, or the specifications of the sonicator (e.g., model, power) and mixing time.	Paste of OPC	Delay in hydration rate	^62^
GO (thickness = 1 nm)	The mixture of GO and PCE was magnetically stirred for 30 s. The stirring rate and other equipment details were not specified.	Paste of OPC/alite/clinker	GO retarded clinker hydration but accelerated OPC and alite hydration	^63^
GO (thickness = 1 nm)	GO was dispersed in deionized water via sonication; however, the specifications of the sonicator (e.g., power, amplitude, model) were not clear in the dispersion step.	Paste of alite	GO accelerated alite hydration	^64^
GO (thickness = 1 nm)	Simply dispersed with a high-speed shear mixer at low speed for 15 s (100–200 rpm)	Paste of OPC	GO accelerated OPC hydration	^65^
GO (particle diameter = 24.6 μm; thickness = 6.9 nm; purity = 99.8%)	Previously dispersed with different dispersants (PCE, NS, MS) with aid of sonicator for 30 min in a probe sonicator with output power of 360 W	Paste of OPC	GO accelerated OPC hydration	^66^

a PCE = Polycarboxylate-based superplasticizer; SC = sodium cholate; SDBS = sodium dodecyl benzenesulfonate; PF127 = Pluronic F127; NS = naphthalene superplasticizer; MS = melamine superplasticizer; OPC = ordinary Portland cement.

According to Adhikary et al.,^59^ who investigated the effects of different-sized GNPs dispersed with PCE through sonication on early hydration of cement mortars, the greater the thickness of the GNPs, the greater the acceleration of cement hydration. For smaller thicknesses, the delay in hydration occurred regardless of the amount of GNPs, hindering the growth of C–S–H.^59^ Additionally, a slight decrease in hydration of GNP-modified cement pastes was reported even with the use of different dispersants due to surrounding water adsorption and trapping by GNPs, resulting in less water for C–S–H growth.^28^ On the other hand, contrasting with the tendency to delay the hydration reaction, Jing et al.^60^ observed only a minimal impact on cement hydration when GNPs were incorporated into cement pastes using dry mixing. Observations indicating a negligible impact of GFMs on cement hydration have also been reported in studies involving the addition of low-oxygen-content GO and conventional graphene with PCE.^61,62^

In turn, the influence of GO on hydration may depend on the composition of the cement matrix, at least in early hydration. GO was found to accelerate hydration in pastes produced only with alite or commercial Portland cement, while having an opposite effect in pastes containing pure clinker.^63,64^ The hydration delay was attributed to ion diffusion hindering by GO, changing the ionic concentration at the liquid–solid interface, potentially via complexation with calcium. The location of GO particles in the cement matrix—either in the pore solution or on the clinker surface—could dictate its effect on hydration: accelerating the reaction in pore solution and acting as an extra surface for the growth of hydrated phases or retarding the process when in the clinker surface. The addition of gypsum was found to reverse the hydration delay due to release of sulfate ions and their adsorption onto aluminate sites in clinker surface,^63^ which changed those surfaces in two possible ways: by making the C_3_A surface negatively charged, thus preventing GO adsorption, or preventing the formation of the amorphous precipitate to which GO is bound, with consequent hydration delay.^63^

The size and number of functional groups on the surface of GO may also play a role in determining its impact on cement hydration. According to Li et al.,^67^ samples containing GO with a higher quantity of functional groups and larger particle sizes exhibited a delayed hydration peak compared to the control, but with a higher peak intensity. However, when analyzing the total heat release at 72 h, it was observed that the quantity of functional groups had a low influence on the overall hydration process. In contrast, an increase in the nanoparticles’ size appeared to accelerate cement hydration, possibly due to the provision of additional growth sites for hydration products.^67^ This suggests that the nanoparticle size rather than functional group density may be a more important factor in promoting hydration kinetics. Despite these findings, GFMs are usually considered as accelerators of the hydration reaction of different cementitious matrices.^67,68^

An increase in the degree of hydration was observed in the samples with GO, evidenced by a 6% increase in the content of the formed Ca­(OH)_2_ compared to the reference samples.^65^ It is discussed that the hydration process is governed by nucleation and growth of hydrated products in GO–cement pastes, which is favored by the presence of GO, with increasing of tensile and compressive strengths of GO–cement pastes by 50% and 46%, respectively.^65^ The observed improvements on mechanical strength and hydration of Portland cement composites reinforced with of GNPs occurred because GNPs accelerated the hydration process and directly influenced the microstructure, leading to the formation of finer Ca­(OH)_2_ crystals as well as higher quantities of hydrated silicates and lower porosity.^66^ After 28 days of hydration, X-ray diffraction analysis revealed that the presence of GNPs increases the intensity of portlandite and ettringite peaks, indicating a better crystallization of these phases.^66^

In summary, the acceleration or delay of cement hydration by GFMs depends on several factors. Thickness around 11–15 nm tends to accelerate the hydration reaction, while smaller ones retard it. The matrix is also critical; GFMs retard hydration in gypsum-free matrices, such as clinker, more than in alite or OPC matrices. Furthermore, GFMs with more functional groups have a greater effect than those with fewer, and effective dispersion is essential for achieving acceleration. Yet, some points still need to be better understood, including how nanoparticles’ composition (including the type of functional groups) and size influence the dynamics during all stages of cement evolution. Moreover, the role of dispersants in mediating GFMs–cement product interactions should be further clarified. The following sections will bring studies describing interaction models between GFMs and cement as well as studies including dispersants.

## 5 Models of iInteraction between GFMs and Cement Matrix

Several processes have been proposed to describe the interactions between the GFMs and cement matrices. One of the most frequently cited interaction mechanisms of GFMs and cement was described by Lv et al.^42^ in a study in 2013. The mechanism was based on the occurrence of reactions involving functional groups of GO with the anhydrous components of the cement, which may create preferential sites for the growth of the hydrated products. According to this model, this reaction would proceed, leading to the formation of hydration crystals resembling thick columns, which could evolve further into flower-like structures (Figure 3).^42^

**Figure 3:**
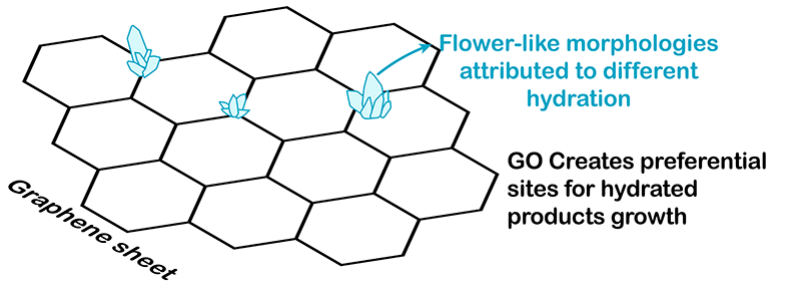
Illustration of the interaction model between the GO and cement.

Although Lv et al.^42^ attributed the emergence of these morphologies to the formation of hydration products resulting from the interaction between cement components and graphene oxide sheets, other studies indicate that the presence and amount of dispersants significantly influence the formation of these crystals, which, in fact, correspond to calcium carbonate.^69,70^ Prudente,^69^ through scanning electron microscopy analyses, observed that this CaCO_3_ morphology forms in cement/PCE, cement/GNP, and cement/GNP/PCE systems. Furthermore, in pastes hydrated for 24 h, those containing PCE and GNP/PCE exhibited a higher degree of carbonation compared to pure cement pastes.

In the same context, Keller and Plank^70^ evaluated the formation of CaCO_3_ in aqueous solution in the presence of PCE. The authors describe that, as the size of the PCE side chains increases, the morphology of CaCO_3_ can vary, giving rise to unusual shapes such as dumbbells, spherules, and flowers. Under high pH conditions, the presence of PCE can lead to a nonclassical crystallization mechanism, in which the chelation of Ca^2+^ ions plays a key role in nucleation. The nanocrystals stabilized by PCE progressively aggregate, resulting in diverse morphologies. This fractal growth, guided by the interaction of PCE with the crystallites, produces highly organized mesocrystals.

In 2019, for GO-modified pastes, Wang et al.^66^ proposed a model based on the formation of a 3D network of GO sheets cross-linked by [COO]^−^[Ca]^2+^[OOC]^−^ bridges, resulting from the interaction between carboxyl groups from GO and calcium ions from Ca­(OH)_2_ during the hydration reaction. Wang^66^ proposed a model based on the formation of a 3D network of GO sheets cross-linked by [COO]^−^[Ca]^2+^[OOC]^−^ bridges, resulting from the interaction between carboxyl groups from GO and calcium ions from Ca­(OH)_2_ during the hydration reaction (Figure 4). In 2016, GFMs were proposed to act as a catalyst for the cement hydration reaction.^71^ In this model, oxygen-containing functional groups on the GO surface would have two primary functions: (1) to act as interaction sites with cement and (2) to absorb water molecules, creating a reservoir and transport channels for water (Figure 5). Consequently, the water adsorbed on the functional groups could interact with the cement phases, leading to the formation of hydrates.^71^

**Figure 4:**
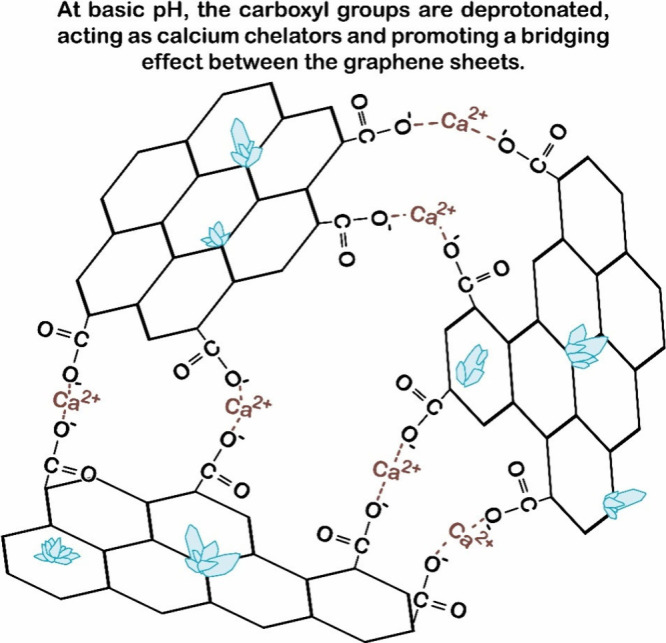
Illustration of the interaction model between GO and calcium ions of the cement.

**Figure 5:**
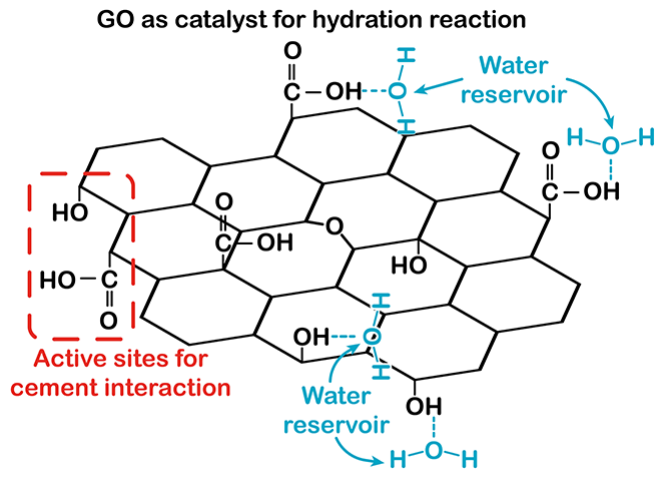
Illustration of the interaction model between GO and water produced from the cement.

Recently, Meng et al.^43^ proposed that calcium ions would initially be adsorbed on the GNPs/GO surface, and, as time progresses, the high ion mobility promoted by GNPs/GO would lead to the formation of ionic clusters, thereby promoting the generation of hydration products.^43^ However, these products would not arise due to the nucleation and growth effect of graphene, but rather due to the increased mobility of ions generated by GNPs/GO, favoring the nucleation and growth of hydration products such as C–S–H on the surface of the cement particles, with a typical needle-like morphology.^43^

Additionally, GO induced the directional growth of C–S–H, resulting in an organized “comblike” structure during the early stages. Once again, although the authors used PCE for GO dispersion, the effect of PCE was not considered in the construction of the GO/cement interaction model or in the cement hydration reaction.^67^ Figure 6 illustrates the interaction between the functional groups of GO and the surface of the cement hydration products.

**Figure 6:**
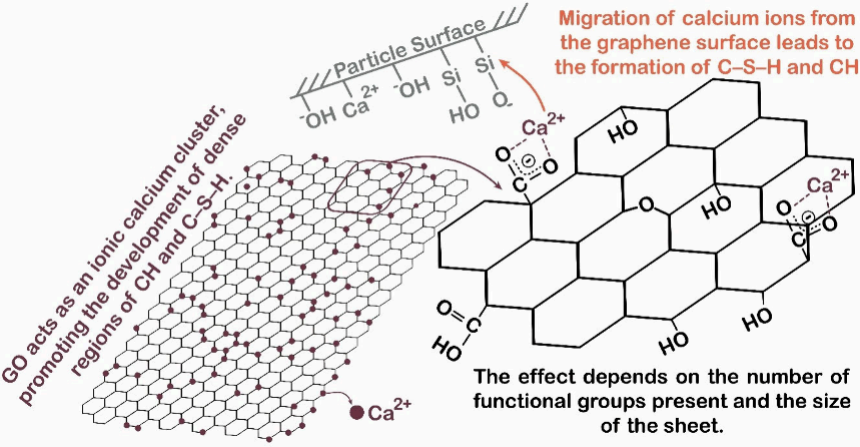
Illustration of the interaction between the cement hydration products and functional groups of GO.

In contrast, Kong et al.^29^ investigated the impact of GO and rGO with varying degrees of reduction, showing that the quantity and morphology of hydration products differed depending on the amount and type of functional groups present on the GO/rGO surface. Samples containing GO exhibited a higher amount of hydration products than the control with a cross-linked and dense microstructure. Additionally, spherical and diamond-shaped morphologies, attributed to calcite and vaterite, respectively, were observed. Samples with moderately reduced rGO showed fewer hydration products and presented pine-needle-like and flower-like CaCO_3_ particles, whereas samples with highly reduced rGO displayed a non-uniform distribution of hydration products, along with cracks and pores.^29^

According to Kong et al.^29^ at the beginning of hydration, rGO with more functional groups initially accelerated the hydration rate, promoting the formation of C–S–H and CH. As hydration progressed, the rGO surface expanded directionally, forming a calcium-rich region (Figure 7). Simultaneously, rGO containing acidic oxygen groups neutralized the alkaline environment, producing CO_2_, CO, and H_2_O, which enhanced the cement dissolution rates and accelerated early hydration. The functional groups of rGO acted as reaction sites for the carbonation of hydration products and the formation of calcium carbonate. As previously mentioned, the morphology of CaCO_3_ varied based on the quantity and type of functional groups in GO/rGO, promoting the development of intercross-linked microstructures and increasing matrix density (Figure 7).^29^ Despite the use of a dispersant to distribute GO/rGO, its influence on hydration and microstructure, as well as its role in nanoparticle/cement interactions, was not considered in the model presented by the authors.

**Figure 7:**
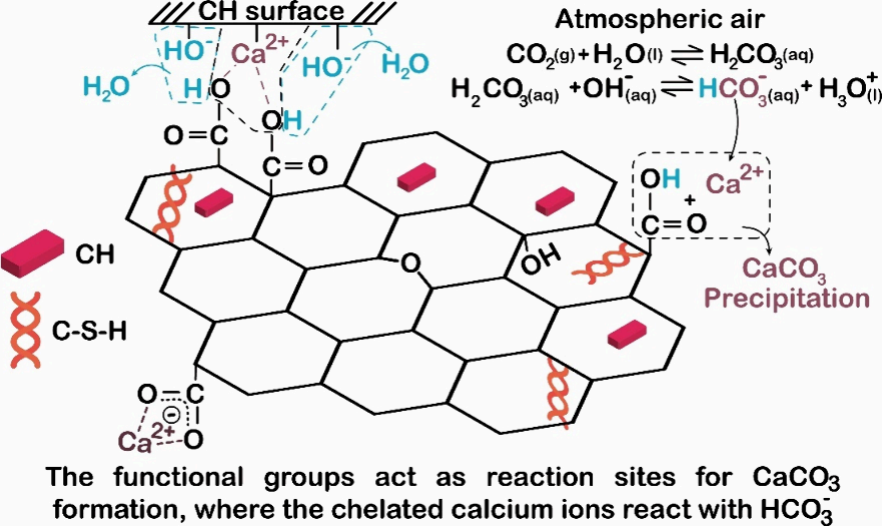
Illustration of the reaction between atmospheric CO and CO_2_ with the -[COO^–^]­[Ca^2+^] groups leading to CaCO_3_ precipitation.

Li et al.,^67^ similar to Kong et al.,^29^ investigated the influence of functional groups and the impact of GO particle size on the cement paste hydration reaction model. According to the authors, in the initial hydration phase, Ca^2+^ ions are attracted by the functional groups of GO, which act as nucleation sites for hydration products with GO playing a regulatory role in the growth of these phases. In samples with GO containing a higher number of functional groups or larger sheets, it was observed that GO regulates the size of CH crystals at early ages, promoting their spatial compaction.

The capture of Ca^2+^ ions on the graphene surface occurs in regions containing the −COOH and −OH groups. During cement hydration in the presence of GO, calcium hydroxide also reacts with the carboxylic groups of GO: the −COOH groups are deprotonated to −COO^–^, which act as ligands for Ca^2+^ ions, leading to the formation of calcium-rich regions with the release of H_2_O. These and other chelating groups are likewise present in most organic dispersants, such as PCE, which interact with the medium and bind to Ca^2+^, thus competing with GO. The model proposed by Kong et al.^29^ presents inconsistencies, as it suggests that this reaction also leads to the release of CO and CO_2_ for subsequent CaCO_3_ formation. Nevertheless, the calcium carbonate formed in the final stage probably results from the uptake of atmospheric CO and CO_2_ in the calcium-rich regions. For Kong et al.^29^ model to be chemically feasible under their experimental conditions, it should be necessary for a a graphene ring cleavage to occur; however, their experiments were not conducted under an inert atmosphere. Therefore, the most probable hypothesis is that CO and CO_2_ would be adsorbed into the paste according to the following reaction sequence:

1 $$2\mathrm{CO}+2H_{2}O\to H_{2}{\mathrm{CO}}_{2}+\mathrm{HCOOH}$$

2 $${\mathrm{CO}}_{2}+2H_{2}O\to H_{2}{{\mathrm{CO}}_{3}}^{-}+H_{3}O^{+}$$

3 $${{\mathrm{HCO}}_{3}}^{-}+R_{1}‐{\left[\mathrm{COO}\right]}^{-}{\left[\mathrm{Ca}\right]}^{2+}\to {\mathrm{CaCO}}_{3}+R_{1}‐\mathrm{COOH}$$

In this equation, R_1_-[COO]^−^[Ca]^2+^ refers to GO with calcium-rich regions resulting from the model (Figure 6). Chemically, under atmospheric conditions, precipitated carbonate originates from the reaction between atmospheric CO_2_ and the pore solution of the cement paste. This reaction is facilitated by GO that has already reacted with CH from the cement.

On the other hand, the dispersant SDBS plays a role in the GNPs/cement interaction mechanism proposed by Baomin et al.,^72^ involving the adsorption of SDBS hydrophilic groups onto the graphene surface. Following hydrolysis, calcium ions are adsorbed onto the GNPs surface coated with SDBS, leading to the generation of C–S–H and AFt as the solution becomes supersaturated with Ca^2+^.^72^ This process would ensure sufficient microchannels for the transport of water retained in the hydrolysis layer of the cement particles, facilitating clinker ion exchange and providing more space for C–S–H nucleation and growth. Gypsum in the clinker would be consumed faster, leading to a reaction between AFt and C_4_AH_13_, producing AFm earlier and accelerating hydration reaction in the early stages. The process continues, with obstruction of the hydrolysis layer by hydration products, decreasing the hydration rate. A more compact microstructure with the presence of AFm was detected at early stages^72^ (Figure 8).

**Figure 8:**
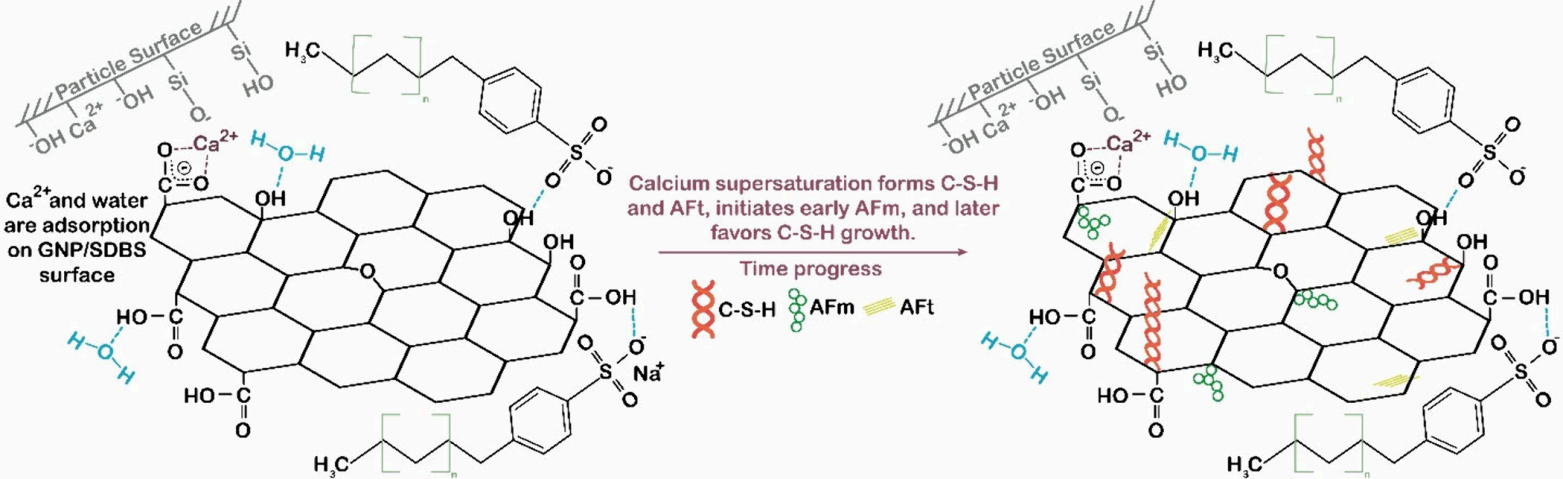
Illustration of the interaction model between GNP, SDBS and cement.

Another method to disperse graphene-based materials (GFMs) is by using nanosilica. In their study, Liu et al.^36^ developed a GO/nanosilica hybrid by first dispersing nanosilica onto the GO surface through covalent bonds formed between the Si–OH groups of the silica and the functional groups of GO. In the cement matrix, this hybrid nanomaterial would act as nucleation sites for hydration products in the cement matrix. The nucleation effect of GO’s functional groups predominates during the early stages, accelerating the cement hydration process. As the reaction proceeds, pozzolanic effect of nanosilica would generate secondary C–S–H through reaction with CH, favoring strengthening of the hardened paste.^36^ Furthermore, electrostatic interactions between nanoparticles favored the growth of the C–S–H gel around the hybrid. The C–S–H from this process then merged with secondary C–S–H forming the network structure based on GO, while the hydration products grew within pores voids densifying the microstructure (Figure 9).

**Figure 9:**
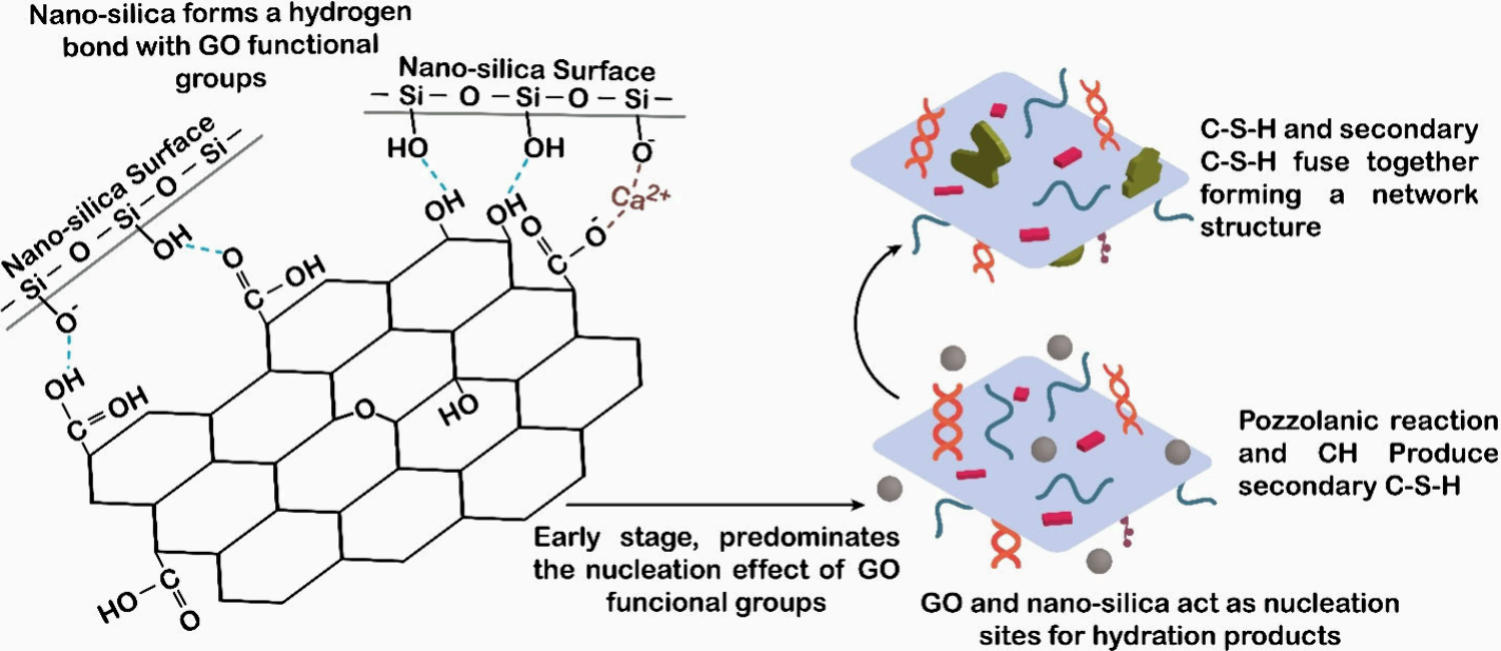
Illustration of the interaction model between GO/nanosilica and cement.

The use of homogenization methods may influence reaction pathways, as investigated by Xiong et al.,^18^ who studied different mixtures of GO, cement, and water, both with and without PCE, using a mechanical stirrer and power ultrasound (PUS). PUS waves led to the partial dispersion of GO, providing additional carboxyl groups for hydrate nucleation and accelerating the dissolution of calcium ions, thereby promoting the formation of C–S–H and CH.^18^ When PCE was added during the mixing process, the main chain was adsorbed on the GO surface and provided steric hindrance between the nanoparticles. Additionally, calcium present between interlayers of C–S–H interacted more strongly with the carboxyl groups from GO when PCE was added. The combination of PCE and PUS inhibits PCE self-agglomeration, and PCE adhered better to the GO surface by increasing the surface area of GO and providing nucleation sites for CH and C–S–H^18^ (Figure 10).

**Figure 10:**
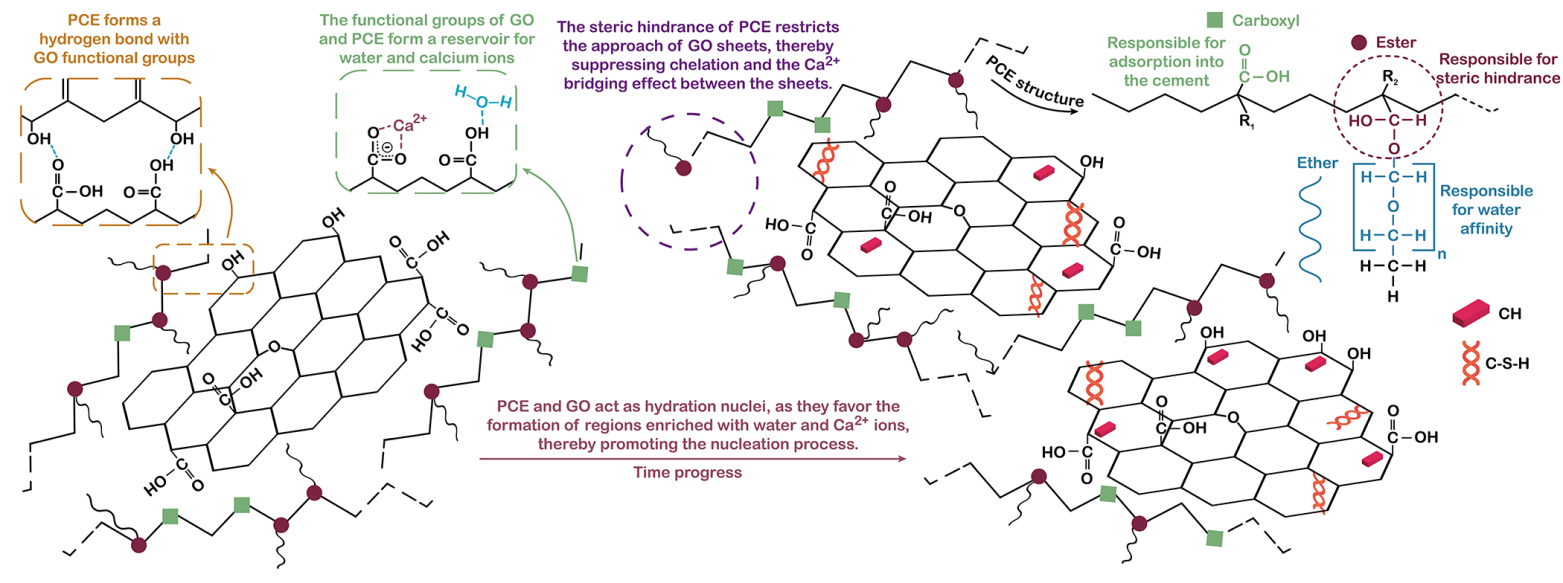
1Illustration of the interaction model between the GO/PCE and cement.

Alternatively, Wang et al.^73^ proposed two models of interaction between the nanoparticles, cement and PCE. In the first mechanism, the rGO would surround the cement particles, forming a core–shell structure, limiting the interactions between adjacent cement grains and the cement grains with their hydrates.^73^ While in the second possible interaction, the PCE would be adsorbed on the nanoparticle surface and would induce the formation of portlandite crystals with large particle sizes on the GO/PCE surface. Both mechanisms would weaken the 3D network of hydration products (Figure 11).^73^

**Figure 11:**
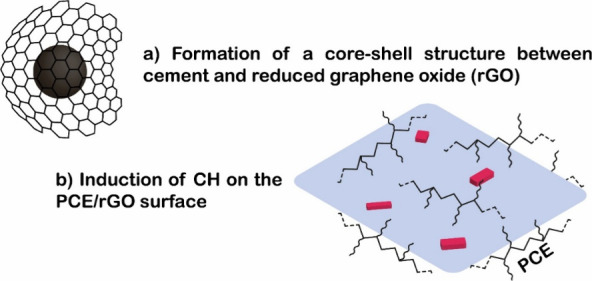
Illustration of the interaction model between the rGO/PCE and cement.

As observed, the lack of consensus regarding the interaction mechanism between GFMs and cement is largely due to the cement system’s inherent complexity and significant variations in experimental conditions. These include the type of cementitious matrices, the GFMs used, and the methodologies for dispersing the GFMs and preparing samples.

This variability hinders a direct comparison between studies and makes it difficult to construct unified interaction models. For instance, while some studies provide detailed information about GFMs characterization (such as thickness, particle size, number of layers, purity, functional groups, and defect density), others offer minimal information, indicating only the type of GFMs used. However, these characteristics are very important for understanding how GFMs influence cement hydration products.

Effective GFMs dispersion is often achieved using dispersants or through covalent functionalization. However, much research neglects the impact of these additives while constructing their graphene–cement interaction models, raising uncertainty about whether the observed effects are truly due to the GFMs or the dispersants themselves. This is particularly relevant since many dispersants used for GFMs (e.g., superplasticizers) are already used in cement sample preparations, known to affect cement properties, such as workability and water reduction. Thus, their influence cannot be overlooked.

Therefore, future studies should provide detailed GFMs characterization and explicitly account for the role of the dispersants. Although standardization is difficult, advancing graphene–cement interaction models requires standardized methodologies. These requirements include the complete characterization of GFMs, detailed reporting of the dispersants, and a systematic study to optimize the dispersion for the specific GFMs used in each study. This type of approach is important for building reliable correlations among material properties, processing methods, and composite performance.

## 6 Effect of GFMs on Workability of Cementitious Matrix

The workability of cementitious materials, which is related to the rheology in their fresh state, can be significantly reduced by the incorporation of nanomaterials into cement.^74^ Owing to their nanoscale dimensions and a large surface area, GFMs demand a high amount of water to wet their surfaces, consequently reducing the amount of free water available to lubricate the cementitious matrix mixture at a constant w/c ratio.^74^ Thus, several factors need to be considered in the presence of GFMs, such as w/c ratio, GFMs content, particle size, presence of functional groups, addition of dispersants, and mixing conditions to enhance particle dispersion (Figure 12).^75^

**Figure 12:**
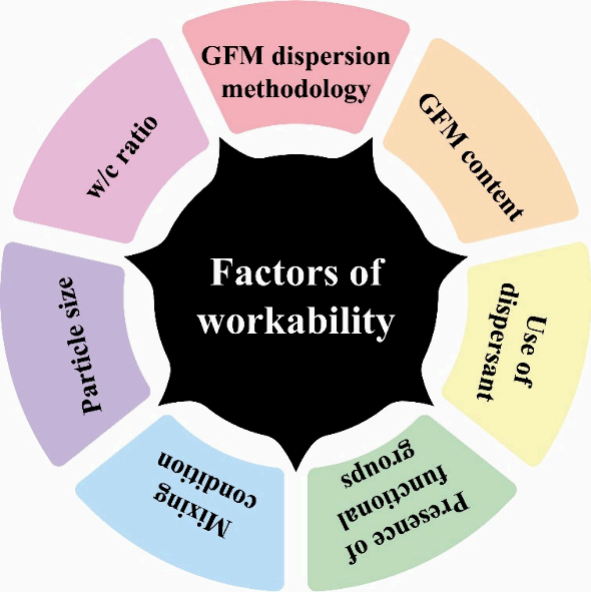
Factors that may affect the workability of cementitious matrices modified with GFMs.

For instance, the decrease in workability resulting from the addition of GO can be attributed to the electrostatic interaction between negatively charged GO and cement particles and leads to aggregate formation and flocculation. Water retention within the flocculated structure increases friction among particles and decreases the fluidity of the paste.^76^ Another explanation for aggregate formation is the presence of divalent ions in the pore solution of the cement paste, particularly calcium, leading to cross-linking of adjacent GFMs sheets.^26^

Also, some studies indicate a distinct behavior of cement matrices modified with GFMs, in terms of workability, depending on the number of functional groups present in the GFMs. According to Qureshi et al.,^77^ cement pastes modified with GO showed lower static flow values compared to samples with rGO or pristine graphene. This effect was attributed to the hydrophilicity of GO, which absorbed water from the paste.

Changes in sample preparation methodologies significantly affect the fresh properties of concrete with the addition of GFMs. For example, Jaramillo et al.,^78^ investigating the addition of 0.25 wt % of GNPs in concrete, varied preparation parameters such as time (5 and 30 min), mixing (hand or drill), and ultrasonication use for GNPs dispersion. Manual dispersion and drill mixing reduced the spread in slump test by 43 and 33.6%, respectively, while ultrasound dispersion reduced workability losses.^78^ Finally, longer mixing times linearly decrease workability for both sonicated and nonsonicated mixtures, evidencing that GFMs dispersion parameters can significantly impact the matrix workability.

The use of dispersants may also improve workability, as superplasticizers are an alternative to preserve workability and enhance particle dispersion within the cementitious matrix, compensating for the reduced available water in the fresh mixture.^42,66,68^ In this sense, PCE is the most used additive, followed by NS, and MS and SDBS.^66,79–81^ Particularly, PCE can effectively induce particle deflocculation and release the water retained, decreasing viscosity by up to 95% and increasing spreading diameter in the mini-slump test.^82^ Evaluations of the effects of GO (0.00–0.04 wt % of cement) were conducted on pastes with a water-to-cement ratio of 0.4 and without superplasticizers, revealing a significant loss of workability.^83^ While the pastes with pure cement exhibited a spread diameter of 57 mm in the mini-slump test, the pastes containing 0.03 and 0.04 wt % GO showed an average spread of approximately 45 mm, representing a decrease of about 21%.^83^ Similar effect was also reported by Gong et al.^65^ and Zhou et al.^84^ Despite this adverse effect, the addition of GFMs may reduce the bleeding rate, as demonstrated by Zhou et al.,^84^ who reported an 82.8% reduction with the addition of 0.05% GO.

Thus, the dispersion of GFMs not only influences the hydration rate but also affects parameters such as workability. Thus, ensuring the effective dispersibility of GFMs is crucial to achieving desirable qualities, as inadequate dispersibility could lead to compromised workability and other undesirable outcomes.

## 7 Effect of GFMs on the Mechanical Properties of Cementitious Matrix

The formation of defects during hydration process and increasing sensitivity to the surrounding environment are inherent traits of cementitious materials and may induce the formation of voids and microcracks, as well as changes in pH and the equilibrium between the constituent phases.^85–87^ Several material’s properties may gradually change, potentially compromising its mechanical strength and durability over time. Given the remarkable attributes of GFMs (such as high surface area, modulus of elasticity, and tensile strength),^74^ extensive research has focused on refining the characteristics of cementitious matrices through their incorporation.

The impact of GFMs on the mechanical properties of cementitious matrices is typically investigated by compressive, tensile, and flexural strength tests. Numerous studies indicate that the addition of GFMs enhances these properties by controlling the morphology of the hydrated products^65,88–90^ and contributing to nanoscale reinforcement of the matrix.^29,42,43^ Porosity reduction is another outcome achieved through the addition of GFMs in cement composites, which not only leads to improvements in mechanical strength and shrinkage control but also influences the durability of the composites.^66,67,84,88,89,91^ A study conducted by Wang and Pang^66^ revealed a significant enhancement in the compressive strength of cement pastes upon modification with GNP, which was assigned to the reduced porosity and to the capacity of GNP to enhance internal friction within the composites under external loads, dispersing concentrated stress and mitigating cracking propagation.

One of the major challenges in using GFMs in cementitious matrices is to define the optimal additive amounts that provide the best performance and durability of those matrices. Many variables significantly influence the results obtained for GFMs/cement systems in different studies, including the type of matrix, the type of GFMs (GO, GNPs, rGO), the size of the nanoparticle, and the methodology used for the GFMs dispersion, as can be seen in Figures 13–16. For instance, Yang et al.^92^ and Long et al.^93^ evaluated the compressive strengths of pastes containing up to 0.20% by weight GO. While Yang et al.^92^ identified that strength increases following the GO content, reaching the maximum strength increase at 0.20 wt % GO, Long et al.^93^ found higher strength increase at 0.10 wt % and lower at 0.20 wt % GO.^93^ In the same sense, Bagheri et al.^94^ and Gholampour et al.,^95^ reported a lack of an optimal value for mortars with GO in concentrations up to 0.5 wt %.

**Figure 13:**
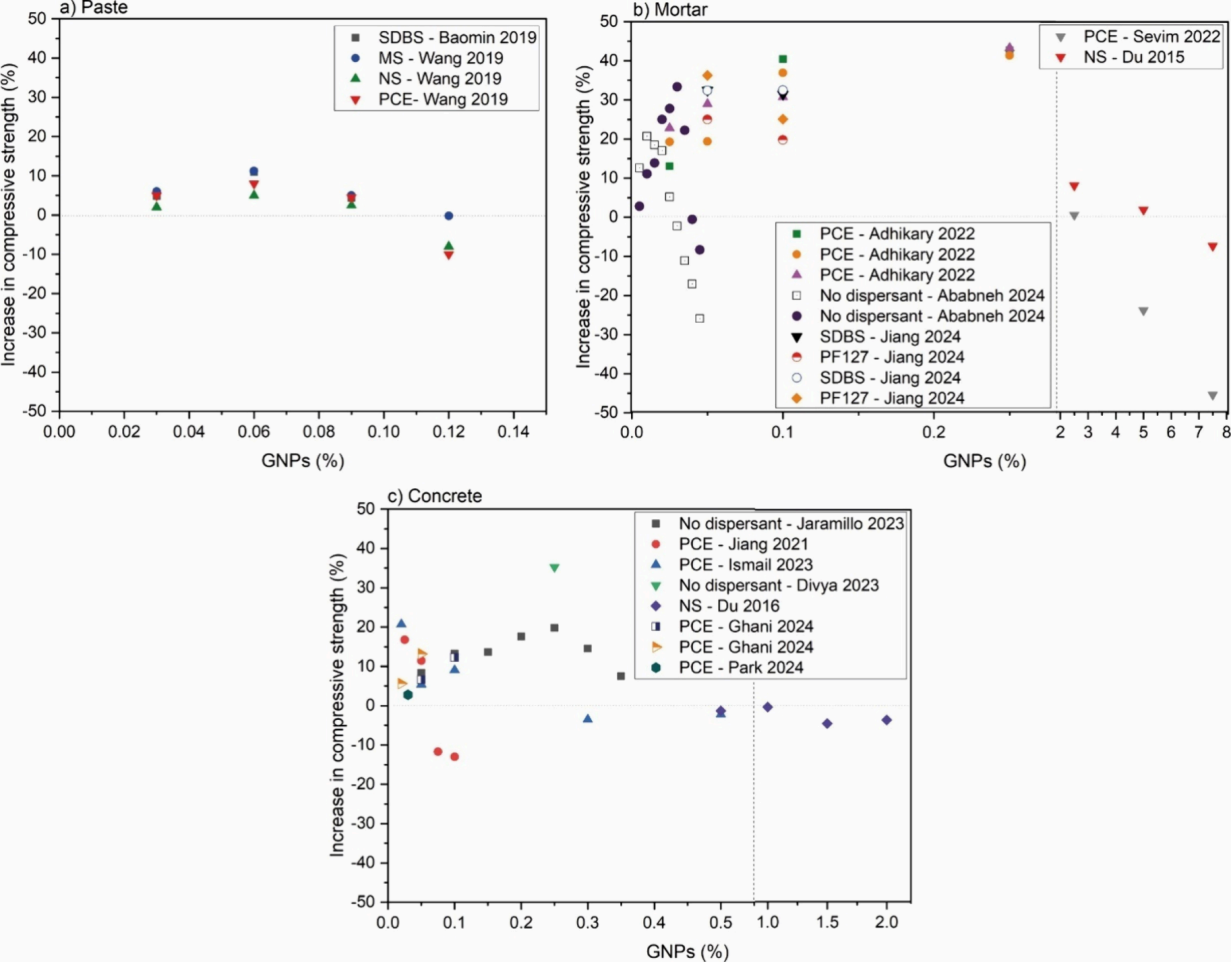
Comparative graph of the compressive strength of (a) pastes,^66,72^ (b) mortars,^25,59,96,98,99^ and (c) concretes^19,44,78,80,97,100,101^ with the addition of GNP. PCE = polycarboxylate-based superplasticizer, NS = naphthalene superplasticizer, MS = melamine superplasticizer, SDBS = sodium dodecyl benzenesulfonate.

**Figure 14:**
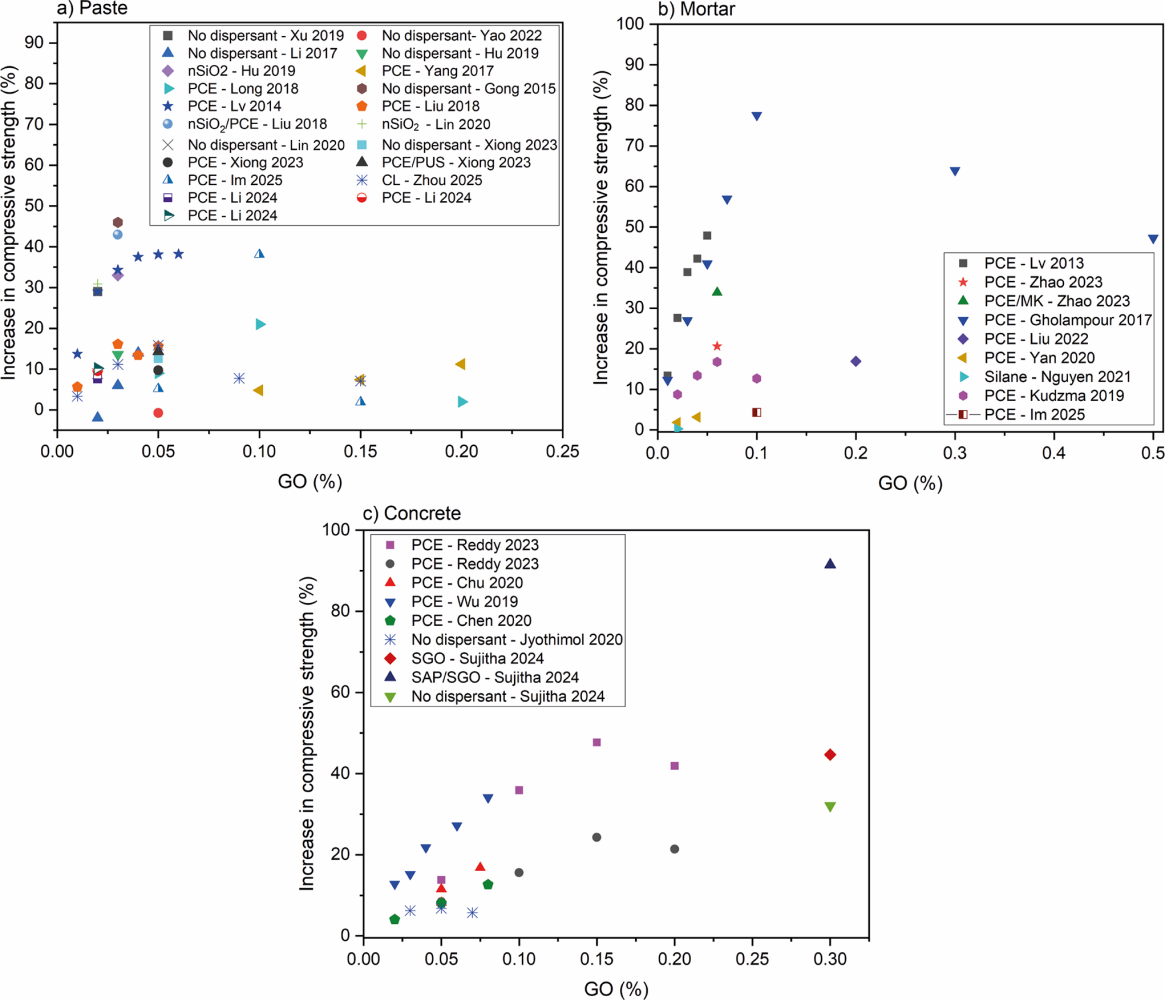
Comparative graph of the compressive strength (a) of pastes,^18,33,36,65,67,68,83,84,88,92,93,102–104^ (b) mortars,^32,42,53,61,68,79,105^ and (c) concretes^1,3,6,106–108^ with the addition of GO. PCE = polycarboxylate-based superplasticizer; NS = naphthalene superplasticizer; MS = melamine superplasticizer; SDBS = sodium dodecyl benzenesulfonate; nSiO_2_ = nanosilica; CMK = coal-bearing metakaolin.

**15:**
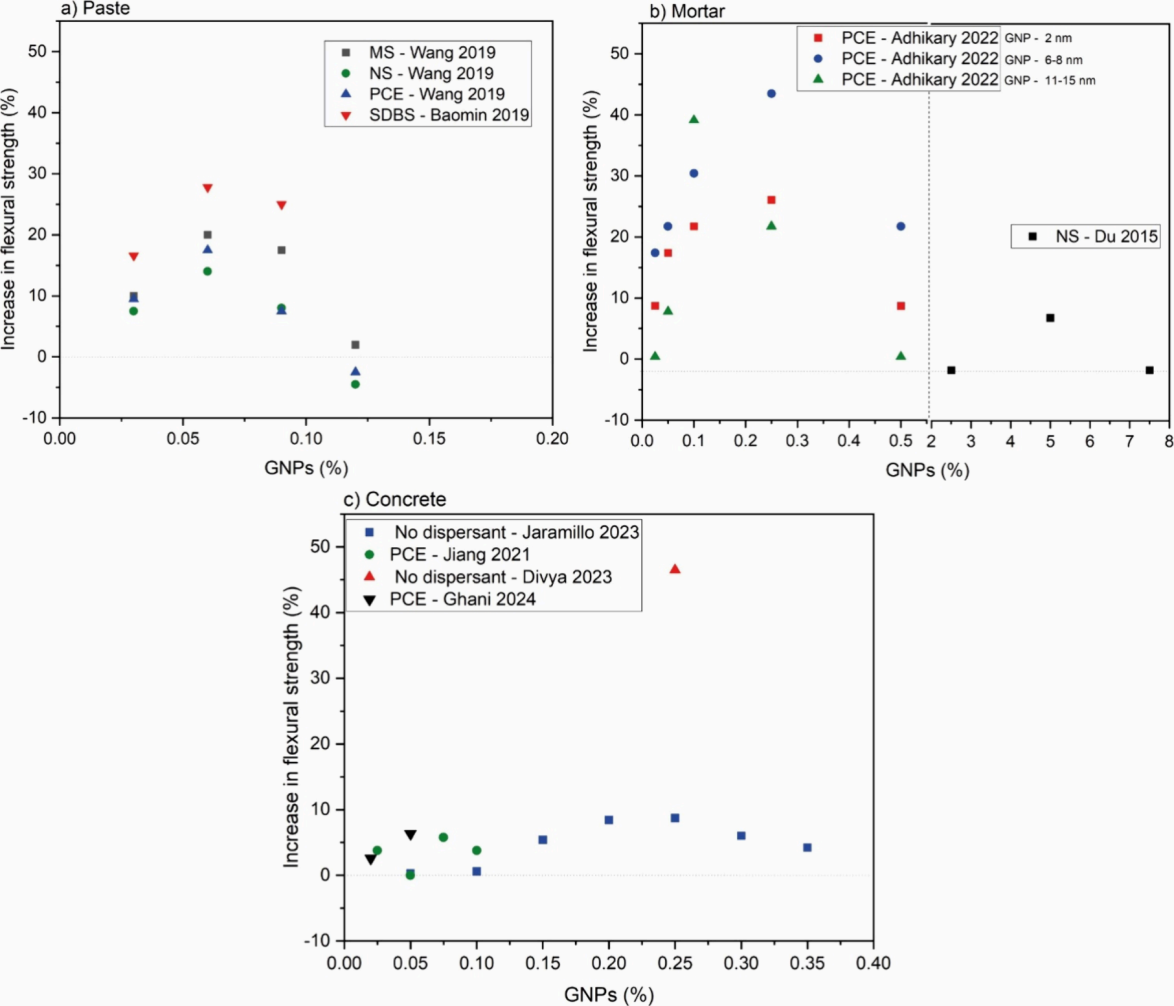
Comparative graph of the flexural strength of (a) pastes,^66,72^ (b) mortars,^25,59^ and (c) concretes^19,44,78,100^ with the addition of GNP. PCE = polycarboxylate-based superplasticizer; NS = naphthalene superplasticizer; MS = melamine superplasticizer; SDBS = sodium dodecyl benzenesulfonate.

**Figure 16:**
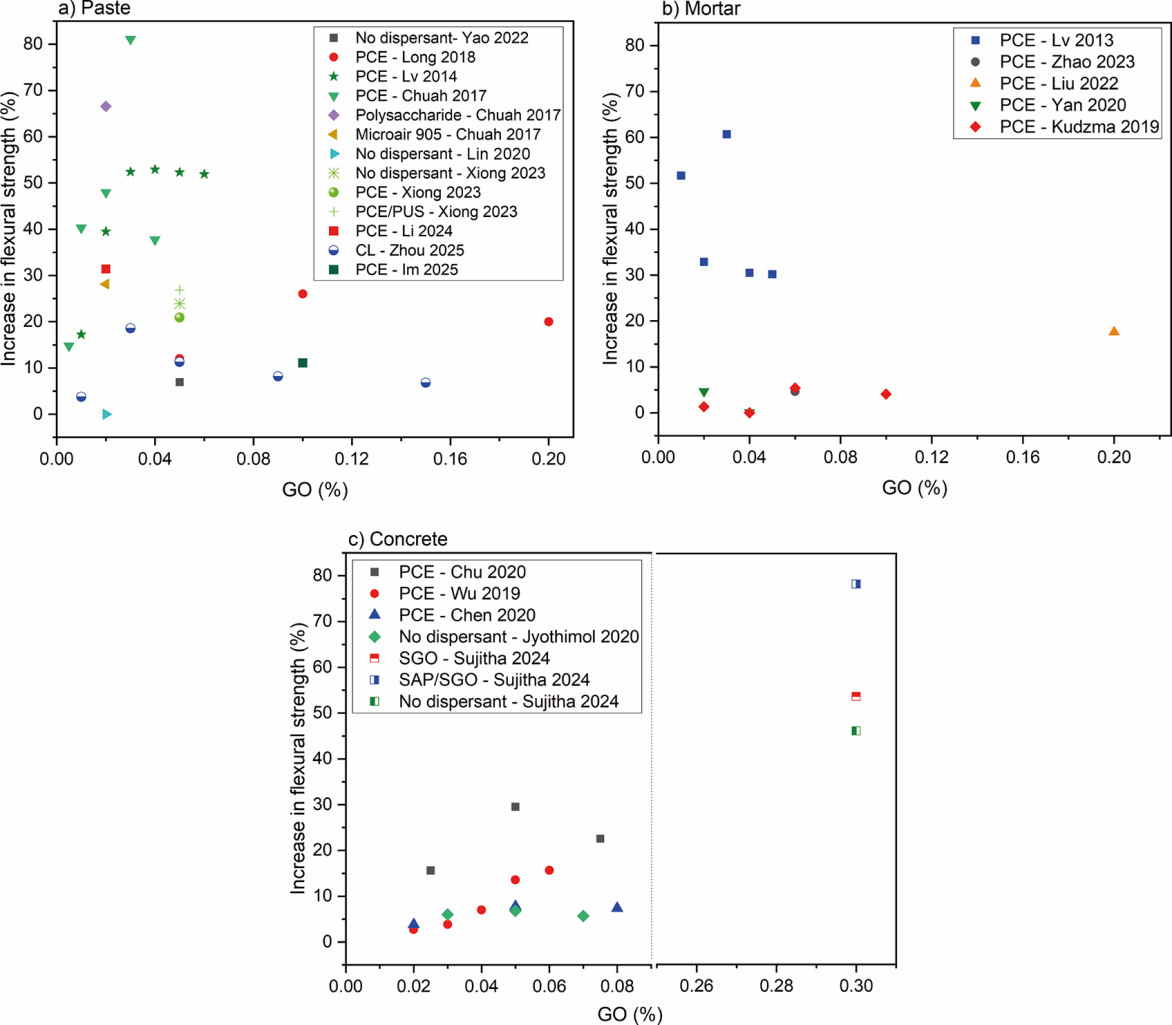
Comparative graph of the flexural strength of (a) pastes,^18,33,67,68,84,88,93,103,109^ (b) mortars,^42,53,61,79,105^ and (c) concretes^1,3,6,107,108^ with the addition GO. PCE = polycarboxylate-based superplasticizer; NS = naphthalene superplasticizer; MS = melamine superplasticizer; SDBS = sodium dodecyl benzenesulfonate.

Regarding the type of cementitious matrix, in general, mortars exhibited superior mechanical properties compared with pastes and concretes. For instance, Figure 14 illustrates this trend, where under similar water/cement ratios, same dispersant and identical GO content, mortars exhibited higher strength gains than pastes.^42,79,88,92,93,95,110^ One possible explanation for this behavior is the shearing effect of sand on GO disaggregation, first proposed by Lu et al.^110^ According to the authors, the shearing force of sand can help break aggregated GO into smaller pieces, and longer mixing times contribute positively to this effect. However, this mechanism has not yet been fully elucidated and requires further investigation to confirm its validity.

Another way to improve the mechanical properties of CBMs involves modifying the interfacial transition zone (ITZ). For example, the addition of multilayer graphene (0.3% by weight) led to an increase of up to 27.8% in interfacial bond strength, attributable to greater fixation of hydration products on the aggregate surface and a reduction in the local water/cement ratio.^111^ Similarly, small amounts of graphene oxide (0.02–0.04%) resulted in denser matrices and less porous ITZs, with a reduction in typical pore size from ≈25 μm (control) to <5 μm, reflecting gains in mechanical properties.^53^

In addition to incorporation into the paste, precoating of aggregates enhances the effects on the ITZ: aggregates coated with GNPs showed a reduction in ITZ thickness of up to 63% (vs 38% when GNP was only mixed into the paste), a 33% increase in ITZ hardness, and a more homogeneous transition between aggregate and paste.^112^ Similarly, coating sand with GO reduced ITZ porosity by 30.8% and its thickness to 3–5 μm (vs 15 μm in the control), resulting in 38% increases in compressive strength and 44% increases in flexural strength at 28 days.^113^ These results indicate that although the addition of graphene to the matrix refines the ITZ and improves mechanical performance, coating concentrates the nanomaterial in the critical mesostructural region, maximizing nucleation, densification, and bonding strength.

The maximum amount of GNPs above which the properties are no longer improved depends on the type of matrix. This amount was found to be 0.06% GNPs per cement weight for pastes,^66,72^ while for mortars and concretes, this occurred at concentrations higher than 0.25 wt % ^19,44,59,78,80,96,97,114^ (Figure 13). This confirms the significant role of sand in the dispersion process of GFMs, as satisfactory results are achieved, even with higher nanoparticle quantities. Another example of how the matrix formulation influences the optimal quantities of GFMs was highlighted by Ababneh et al.^98^ The authors observed that mortars with different levels of cement replacement by natural pozzolana showed variations in the ideal amounts of GNPs. For 40% replacement, the best mechanical performance was achieved with 0.03 wt % GNPs, while for 60% replacement, the optimal performance occurred with only 0.01 wt % GNPs. According to the authors, at higher replacement levels, larger amounts of GNPs tend to cause nanoparticle agglomeration, negatively affecting the performance of the samples.^98^

Similarly, GO exhibited a trend in the data, with peak compressive strength improvement observed at specific addition amounts: 0.03–0.05% for pastes,^33,36,65,83,93,102–104^ 0.1% for mortars,^32,42,53,61,79,95,105^ and 0.15% for concretes^6,106,107^ (Figure 14). However, likewise for the GNPs results, it is important to note that while these values represent the general tendency derived from the graphical analysis, there are outliers and variations that deviate from this trend.

Also, the addition of GNPs above 1% showed no impact or even a negative impact on compressive strength due to nanoparticle agglomeration in mortars and concretes. Nonetheless, Sevim et al.^96^ demonstrated that the agglomeration effects of GNPs can be mitigated with naphthalene-based superplasticizers, reducing resistance losses in such samples. This result is promising, as it allows the incorporation of larger amounts of nanoparticles in cementitious matrices, potentially benefiting self-sensing and self-heating systems, which typically require higher GFMs additions.

Among different GFMs, GNPs exhibited a more consistent behavior in different studies, independently of the dispersion methods, compared to GO. The inconsistency for GO may be linked to its higher variability of functional groups in terms of the nature and number and its dependence on the production method, all impacting the dispersibility.^115^ For both compressive strength and flexural strength, cementitious matrices modified with GO showed superior performance (Figures 14 and 16), possibly due to the enhanced interaction between GO and the dispersants improving the nanoparticle/cement interaction.

According to Figures 13 and 16, the incorporation of dispersants generally improved the performance of the samples. However, an exception occurred for GNPs-modified concretes, in which specimens modified with additives exhibited lower performances compared to those dispersed only via sonication without additives (Figure 13c). While PCE remains the most employed dispersant, the responses of the samples in both compressive and flexural strengths varied significantly from works reporting similar systems. This variability is evident in Figure 14a, where the degree of enhancement in compressive strength fluctuates significantly across the literature data compiled for equivalent amounts of GO.^36,42,65,88,104^ This may be attributed to differences in the chemical structure of PCE, which change the interactions with nanoparticles. Furthermore, superior mechanical responses were observed for pastes modified with GNPs and dispersed with SDBS and MS compared to those employing PCE. Additionally, in the case of pastes with GO additions, samples containing nanosilica for GO dispersion exhibited a 43% increase in compressive strength when compared to the control samples.^36^

Figure 17 presents the variation in the percentage increase in compressive strength as a function of the GFMs content by cement weight, compiled from literature data (Table S.2, Supporting Information). The data points are distinguished according to the type of dispersant used in each study. It is important to note that for this analysis data from different types of GFMs and from different cementitious matrices were grouped together.

**Figure 17:**
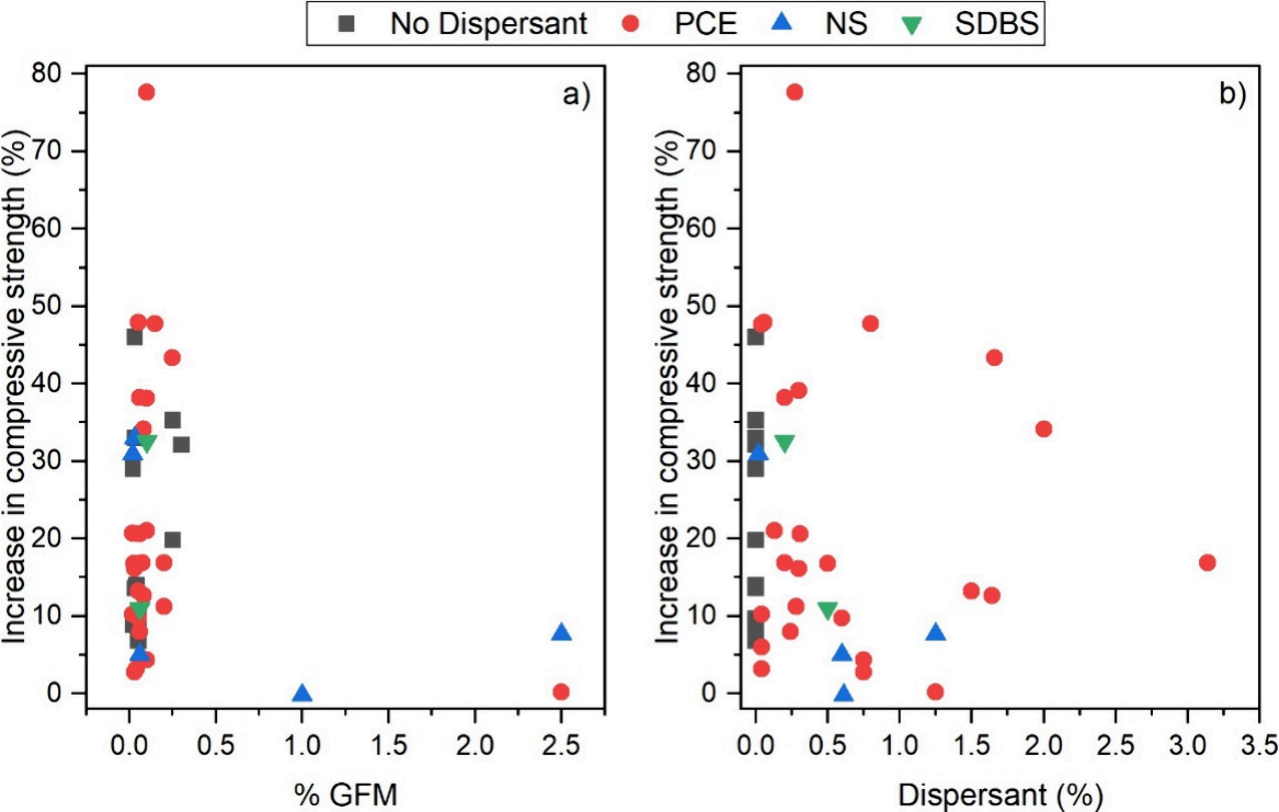
Correlation between (a) percentage of GFMs and increase in compressive strength. (b) Percentage of dispersant and increase in compressive strength.

Overall, it is observed that the highest strength increases are concentrated between 0 and 0.25% of GFMs for samples using PCE or even no dispersant. However, an analysis of variance (ANOVA) performed to evaluate the isolated influence of the dispersant type resulted in a *p*-value of 0.739 (PR­(>F)). This value, significantly above the conventional significance level (α = 0.05), indicates that the observed differences in strength are not statistically significant and cannot be attributed to the “dispersant type” factor in isolation.

When analyzing the correlation between GFMs content and the strength increase in Figure 17a, it is possible to observe that the best mechanical responses occur for a GFMs quantity between 0 and 0.25% by cement weight. Similarly, a second ANOVA was conducted to investigate this correlation, yielding a *p*-value of 0.469, which again does not show any significancy, suggesting the absence of a statistical correlation for this variable within the analyzed data set.

This lack of correlation can be attributed to the high intrinsic variability of the compiled data, which group different types of GFMs (such as GO, rGO, and GNPs) and different cementitious matrices, masking potential specific trends. Different GFMs have distinct surface areas and functional groups, while different matrices offer diverse hydration environments. These factors, combined, can introduce statistical noise that outweighs the signal of the GFMs dosage in the compiled results, reinforcing previous analyses that had already pointed out the complexity of optimizing these nanocomposites. Additionally, a limitation for the analysis is the fact that most studies are focused on this specific dosage range, creating an absence of doses that are not explored, which may contribute to the result dispersion.

In contrast, Figure 17b demonstrates the influence of the dispersant quantity (regardless of its type) on the increase of strength. The data suggest a trend of better mechanical response when the dispersant dosage is in the range of 0–0.5% by cement weight. This time, the ANOVA returned a *p*-value of 0.049, indicating there is a weak to moderate significant difference. Therefore, it is plausible to state that the dispersant quantity has a statistically significant influence on the compressive strength increase, with an optimal dosage likely existing within the identified range. It is important to emphasize that this value is very close to the significance threshold (α = 0.05), suggesting that the influence of the dispersant quantity, although detectable, may be sensitive to the inclusion or exclusion of a few data points. Future analyses with a broader data set that discriminates between GFM types, GFM sizes, and cementitious matrix types are necessary to consolidate this conclusion.

Nonetheless, these results suggest that the main role of the dispersant in this context is to ensure a homogeneous distribution of GFMs within the matrix, an effect that appears to be more dependent on the adequate dosage than on the specific chemistry of the dispersant if it is functional. Future studies that rigorously control the type and characteristics of the GFMs, in addition to the type of cementitious matrix, are needed to untangle more specific and robust correlations between GFMs/dispersant dosage and mechanical performance.

Finally, it is concluded that the definition of the optimum content of GFMs is intrinsically dependent on the experimental system. Factors such as the w/c ratio, particle dimensions and functionalization, and dispersion methodology act concurrently on dispersion, nucleation, and agglomeration tendency. At high contents, agglomeration can generate local defects in the ITZ and compromise performance gains, which explains the existence of different optimal incorporation ranges reported in the literature. Therefore, it is essential that studies describe these key parameters in detail in order to enable adequate comparisons and identification of the most appropriate GFM contents to maximize performance.

## 8 Effect of GFMs on Cement Matrix Durability

The addition of GFMs to cementitious matrices can significantly improve their durability, setting aside money for future repairs and reducing waste. The durability gains resulting from the addition of GFMs are a direct consequence of the modifications these materials make to the microstructure of cementitious matrices.^116–118^ When properly dispersed, GFMs act on three main aspects to mitigate the main degradation mechanisms of these matrices: (1) reducing porosity and refining pore size,^116,117^ (2) physically blocking crack propagation,^119^ and (3) offering new capabilities to the systems, such as self-cleaning^8^ and self-healing,^120–123^ by, sometimes, altering the phases formed in the matrix.

Many studies report reductions in permeability, sorptivity, and chloride penetration in cementitious matrices modified with low dosages of GFMs, often as low as 0.02% by weight of cement (Table 3). For instance, the addition of 0.2% GO dispersed with PCE decreased the relative permeability coefficient by approximately 96% and reduced water penetration depth by 34% compared to the control sample.^118^ While Zhou et al.^84^ were able to decrease the permeability height by 78% and relative permeability coefficient by 96%, with even less amount of GO, 0.05% GO by mass of cement. This shows us that reduction of the permeability of these materials can be achieved with very low amounts of GFMs when associated with good dispersion methodologies.

**Table 3:** Relationship between Durability Parameters, Porosity, and Dispersion Methodology^a^

Matrix type	Type of GFMs	Best GFMs amount (%)	Dispersion methodology	PR (%)	WPR (%)	RID (%)	Porosity param	AI	Ref
Paste	GO	0.06	GO was sonicated in water to improve its dispersion and then added to cement, along with PCE at 1% by weight of cement.	80	55.5	–	Porosity decreased by 26.35% and the critical pore diameter by 37.69%	–	^116^
Paste	GO	0.06	GO was sonicated in water to improve its dispersion and then added to cement, along with PCE at 1% by weight of cement.	41	23.2	–	Porosity decreased by 11.98% and the critical pore diameter by 37.69%	–	^116^
Concrete	GO	0.2	GO was dispersed in water via 1 h sonication. Then, PCE was added at 0.65% by weight of cement and mixed for 2 min at 2000 rpm. Finally, this GO/PCE dispersion was incorporated into the concrete mixture.	96.69	34.5	–	Critical pore diameter decreased by 54.17%	–	^118^
Mortar	GO	0.02	PCE, at 0.88% by weight of cement, was first dissolved in water. Subsequently, GO was added, and the mixture was subjected to 15 min of simultaneous mechanical stirring and ultrasonic dispersion (with an amplitude of 30–50 μm). This final dispersion was then mixed into the mortar components.	–	39.4	–	Average pore diameter decreased by 11.11%	38.1% shrinkage reduction	^130^
Mortar	GO	0.03	The prewetted recycled fine aggregates (RFA) were first manually agitated for 5 min; then, the RFA were immersed in a GO solution for 24 h to allow GO adsorption onto the particles. This final dispersion was then mixed into the mortar components.	–	–	25.4	Porosity was not quantified; however, SEM observations indicated a clear reduction	–	^131^
Paste	HPG	0.1	GO, water, and PCE sonicated by 30 min. This final dispersion was then mixed into the mortar components.	–	–	24.1	Porosity decreased by 13.0%	–	^117^
Paste	GNPs	0.02	GNS were dispersed in water with CO890 via 30 min sonication before the final dispersion was mixed with cement.	–	–	41.6	Porosity decreased by 39.0%	–	^132^
Mortar	TGI	0.1	TGI, water, and F-127 sonicated by 30 min. This final dispersion was then mixed into the mortar components.	–	–	–	Porosity decreased by 24.7%	–	^125^
Mortar	PSCG	0.03	PSCG was dispersed in water via ultrasonication and then mixed with the other mortar components.	–	–	20.5	Porosity decreased by 15.41%	Reduced crack propagation	^119^
Paste	GO	0.2	An aqueous GO suspension was prepared by magnetic stirring in deionized water for 30 min, followed by 2 h of sonication. Half of this dispersion was mixed with PC. Cement was then added and mixed for 1 min. Finally, the remaining dispersion was incorporated and mixed for an additional minute.	–	–	–	A reduction in porosity was observed	41.2% carbonation depth reduction	^126^

a Obs: PR, Permeability reduction; AI, additional information; WPR, water penetration reduction; RID, reduction in ion diffusion coefficient.

In another study where the main variable was GO size and thickness, the aspect ratio (diameter-to-thickness ratio) proved to be important in determining which GO type most effectively enhances water resistance.^116^ Cement pastes with larger diameters and thinner GO achieved the greatest reduction in both relative permeability and water penetration depth. These improvements were attributed to lower total porosity, smaller critical pore diameter, and reduced average pore size.^116^ Regarding sorptivity, which is an indicator of how quickly a matrix absorbs water and thus its susceptibility to aggressive agents like chlorides or sulfates, capillary sorptivity seems to decrease with increasing GO content.^118^ According to Safarkhani and Naderi,^118^ this effect occurs due to nanoparticle-induced pore refinement and reduced penetration volume.

These improvements are not coincidental; they stem from the changes that GFMs addition makes in the microstructure: increasing tortuosity and reducing the pore diameter. Well-dispersed GFMs act as physical barriers, delaying crack development through crack-bridging, filling micropores, and reducing total pore volume.^117,119^ This disrupts pore connectivity, hindering the migration of water, chloride ions, sulfates, and gases, minimizing the potential for corrosion and degradation, and ensuring superior performance in aggressive environments.^117^ For example, in concrete, chlorides are blocked by the presence of aggregates during diffusion, needing to diffuse around the aggregates in already hardened pastes and in interfacial transition zones. Ion diffusion occurs primarily through microcracks and capillaries (these are the main channels), and the diffusion rate of these ions correlates with channel length.^117^

The longer the channel, the longer the diffusion time. In this case, graphene increases the transport path for chloride ions, as it is difficult to traverse dense graphene sheets, requiring them to bypass each other, which increases the path length, or in other words, the tortuosity of ion transport.^117^ For example, the work of Ying et al.^117^ demonstrated by MIP (mercury intrusion porosimetry) a 14% reduction in capillary porosity (pores with diameters between 30 and 10000 nm) with the addition of 0.1% GFM by weight of cement, which directly corroborates their findings of a 24.06% reduction in cement diffusion coefficient. Similarly, according to Zhao et al.,^124^ the addition of a small amount of GO (0.03% by weight of cement) was able to reduce the chloride diffusion depth by 28%.

Other factors that can also be improved by GFMs addition are the resistance to sulfate attack and carbonation of concretes^4^ and the reduction of shrinkage.^108^ According to Chen et al.,^125^ who developed mortars modified with turbostratic graphene intercalates (TGIs) and oxygen-functionalized carbon nanocoils (O–CNCs), the addition of TGI reduced sulfate expansion by 46.15% after 56 days and mass loss by 56.25% compared to the control sample. Even better results were achieved in samples containing both nanoparticles, with the sulfate expansion reduced by 64.1% and the mass loss reduced by approximately 72%. Promising results were also observed regarding the sulfate attack resistance of the modified mortars.^125^

The authors attribute the reduction in deleterious effects from these aggressive agents to several mechanisms, including (1) nanoconfinement (reduced ion mobility due to pore densification), (2) ionic gating (modified matrices regulating ion flow and thereby reducing the penetration of chlorides and sulfates), (3) physical barrier formation (TGI forming tortuous two-dimensional layers that hinder diffusion), (4) chemical interactions (functional groups on the O–CNCs reacting with aggressive ions and limiting their mobility), and (5) microstructural refinement (total porosity reduced by up to 37% and average pore diameter decreased from 32.5 to 18.6 nm).^125^

In the case of carbonation, the addition of 0.2 wt % GO was shown to reduce the carbonation depth by up to 41.2%.^126^ According to the authors, the presence of GO mitigates the carbonation process through several mechanisms. First, it enhances the production of hydration products, leading to a denser cementitious matrix.^126^ This densification hinders the initial step of carbonation: the dissolution of CO_2_ within the pores. Furthermore, GO impedes the dissolution of portlandite (CH) and the decalcification of calcium silicate hydrate (C–S–H). Beyond promoting the formation of standard hydration products, the study indicates that unique GO/C–S–H and GO/CH composites are formed. Also, those hydration phases are subsequently coated with a carbonated layer, which acts as a protective barrier that increases resistance to carbonation by obstructing the penetration of CO_2_.^126^ In the case of drying shrinkage, when added at optimal dosages and with adequate dispersion, GFMs can effectively reduce the shrinkage. They act by filling pores and capillaries within the cement matrix, which restrict the movement of water and thus mitigates contraction. Furthermore, their pore-refining effect contributes significantly to the overall reduction in shrinkage.^127^

Another property of cementitious matrices that can be enhanced through the addition of GFMs is their resistance to freeze–thaw cycles. The mechanism behind this improvement is similar to the mitigating effect GFMs exhibit against aggressive agents: their ability to refine the matrix microstructure and reduce pore size.^128^ As a result, GFMs act as barriers to the movement of liquid water during freezing, thereby reducing the hydrostatic pressure generated by the expansion of water as it turns into ice. Furthermore, the laminar structure of GO hinders the nucleation and growth of ice crystals, extending the material’s service life when subjected to extreme thermal cycling.^128^ This effect was demonstrated by Zeng et al.^128^ and Zhang et al.,^129^ who produced matrices with the addition of GO dispersed in SDBS + PCE and only PCE, respectively, achieving increases in freeze–thaw resistance of 18.9 and 41.9% compared to the control samples.

In addition to the effects deriving from matrix refinement due to reduced porosity, the mechanical reinforcement, previously discussed, is indispensable for the durability of cementitious structures modified with GFMs. Among other benefits, this reinforcement mitigates the initiation and, more importantly, the propagation of microcracks, preventing cracks from connecting and forming preferential pathways for aggressive agents.^119^

In this sense, Ying et al.^119^ reported that GFMs can act as crack propagation mitigators, primarily by reinforcing the matrix through a higher density of hydration products and second by obstructing crack development paths, thereby requiring additional energy to displace the GFMs and enhancing the matrix’s crack resistance. This occurs because the graphene-modified samples exhibit greater stiffness under increasing stress, display less plastic deformation, and inhibit crack growth.^119^ The authors emphasize that such effects are only achievable with excellent dispersion of graphene nanoparticles, making the study of dispersion methods an essential step. This concern is unanimously evident in all methodologies presented in Table 3, whether through simple dispersion using conventional superplasticizers like polycarboxylates or via more sophisticated treatments, such as functionalization.

The durability of the materials can be further enhanced by imparting new characteristics to these systems. Due to the antimicrobial properties of GFMs, materials modified with these compounds can acquire self-cleaning properties, reducing the detrimental effects of microbiologically influenced corrosion.^8^ Additionally, the self-healing capacity of the materials can promote their durability by amplifying the natural self-healing capacity of cementitious products. In a study evaluating crack closure, cement pastes containing superabsorbent polymers (SAPs), GO, and multiwalled carbon nanotubes (MWCNTs), each at a concentration of 0.1% by weight, were compared.^121^ While pastes containing only SAP showed partial crack closure, those with nanoparticles achieved 100% crack healing after 90 days. Similar studies on silane-modified GO (SGO) and SAP in cement-based materials have proposed the SGO/SAP combination as a possible solution for self-healing applications.^108^ According to the authors, while SAP enhances water retention for the cement matrix, SGO’s hydrophilic groups attract and redistribute water molecules, thus ensuring uniform hydration, promoting secondary reactions, and enabling self-healing.^108^

Therefore, the presence of GFMs enhances the matrices’ capacity to undergo autogenous healing through the hydration of unhydrated residual cement, as hydrated silicates typically form the crack-sealing products.^120–122^ Another explanation for the GFMs self-healing behavior comes from research that identified calcium carbonate in crack closure.^123,133^ In this case, the incorporation of GFMs, especially GO, improves the adsorption of CO_2_ in cement-based materials and ion diffusion during hydration deceleration. The crystals produced by the carbonation reaction aggregate into a flower-like morphology due to the template action of GO. These crystals continue to form and grow over time, until carbonation self-healing reaches the final stage (14–28 days), with almost complete crack closure.^123^ Similar outcome was achieved by Göçügenci and Keskin using GNPs and PVA, with complete crack healing for the GNPs-treated samples.^120^ The described mechanism was also based on the nucleation effect caused by GNPs, enhancing the formation of hydrated products, as depicted in Figure 18.^120^

**Figure 18:**
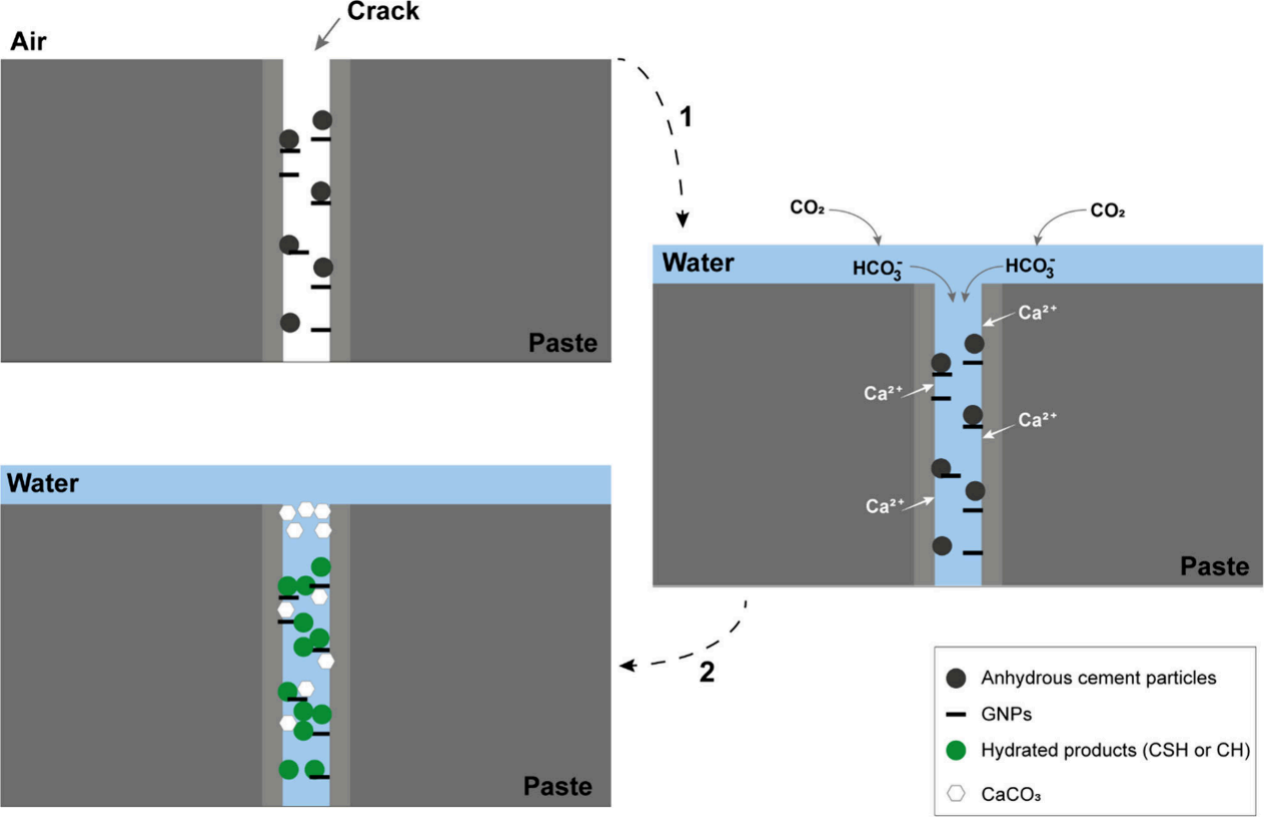
Mechanism of crack closure in composites containing GNPs

## 9 Challenges and Future Perspectives for GFMs Interactions with Cement Matrices

### 9.1 Effect of GFMs on Hydration Rate and Construction of Interaction Models

The complexity of cement hydration makes investigating the influence of GFMs challenging. Different matrices, including pastes, mortars, and concretes, behave differently in the presence of additives, such as GFMs and superplasticizers. This diversity of behaviors reflects the lack of consensus regarding changes in the hydration occurrence, microstructure, and interaction models between GFMs and such matrices. This difficulty is further intensified by the absence of detailed information from the authors regarding the chemical and structural structures of the GFMs and the dispersants used. As a result, it becomes challenging to comprehend the true effects of each component within these systems and compare findings across different studies. Thus, there is still a gap in understanding how the composition of the matrices and the type of nanoparticles influence the dynamics of hydration occurrence during all stages of cement evolution.

Also, simplifications are observed in models describing the interactions of GFMs with cementitious matrices. In some studies, GFMs/cement interaction models are based on systems where GFMs were previously dispersed with a dispersant or functionalized with another material, yet these modifications are not considered in the interaction model. While devising a single model becomes unfeasible due to the complexity of the studied systems, the existing models fail to account for the dispersant and its role.

Hence, some points still need to be better understood, including (1) the influence of GFMs on cement hydration and whether each type of GFM affects hydration differently, (2) how different GFMs interact with cement and the role of dispersants in influencing this interaction, and (3) the impact of GFMs on the morphology of hydration products and whether they promote calcium carbonate formation, for instance.

### 9.2 Impact of GFMs on Cement Workability

The use of GFMs tends to reduce the workability of cementitious matrices due to their ability to retain the water present in these matrices when attempting to maintain the same w/c ratio as in a conventional matrix. However, some strategies can mitigate this problem, such as improving the dispersion processes of GFMs. For this purpose, classical products from the construction industry, such as PCE, can be used. Additionally, other parameters may also influence workability, although their functioning is not yet fully understood. Among these factors are the quantity of functional groups present in the GFMs, the size of the sheets, and the number of structural defects.

### 9.3 Effect of GFMs on Mechanical Properties and Durability of Cement Matrices

#### 9.3a Effect on Mechanical Properties

The works reviewed here highlight the need for a meticulous analysis of each system in which GFMs are employed, emphasizing the importance of selecting the appropriate dispersant and its optimal amount as well as carefully controlling dispersion conditions, since all these factors are extremely relevant. Also, although PCE is one of the most used dispersants, other materials have shown promising results for GFMs dispersion and improvement of mechanical properties, including SDBS and functionalization of GFMs with nanosilica. Further studies should be carried out to better understand the interaction between these dispersants and cement.

Concerning mechanical properties, the cement matrices can be enhanced by the addition of GFMs, yet this effect appears to exhibit significant variability. Discrepancies were noted across different dispersion methods as well as among the various types of cementitious matrices employed. For instance, GFMs demonstrated a more pronounced positive effect on compression and flexural strengths in mortars and concretes compared to cement pastes. Also, this review further highlighted that the impact of GFMs on properties, such as the mechanical strength of matrices, is largely influenced by their chemical and structural characteristics. In many cases, this aspect was more significant than the actual quantity of nanoparticles incorporated.

#### 9.3b Effect on Durability

Furthermore, the use of GFMs can also serve as a potential solution for addressing construction pathologies such as biodegradation caused by microorganism colonization or concrete cracking. However, the literature in this area remains at an early stage, lacking in-depth studies on critical aspects, such as the minimum amounts of additives required to impart these properties. Additionally, factors such as improving the resistance of these concretes to aggressive environments and enhancing their durability still require further investigation.

## 10 Conclusions

GFMs can either accelerate or retard hydration, depending on their type and degree of dispersion. GFMs combined with SDS exhibit a higher tendency toward agglomeration, and agglomeration leads to delayed hydration, mechanical resistance decreases, and durability issues, whereas PCE provides more effective dispersion due to the nature of its chemical interactions and consequently accelerates both hydration kinetics and carbonation. Ultrasonic dispersion improves the workability and homogeneity of the system. The alkaline pH promotes GO agglomeration, underscoring the need for dispersants, which typically interact with GO through van der Waals forces. Although the wide range of experimental conditions prevent robust modeling of the dispersant effect on GFMs dispersion, our analysis suggests an optimal graphene-to-dispersant ratio between 1:1 and 1:4. Nevertheless, even in the absence of a linear correlation between dispersion methods and the use of graphene in cementitious systems, the main effects are associated with hydration control, porosity reduction, and durability enhancement. Optimizing dispersion ensures the homogeneous distribution of GO within the cement matrix and promotes hydration through H_2_O release during the CH/GO reaction, ultimately resulting in improved mechanical strength of the cementitious matrix.

## Supplementary Material



## Notes

(Table S.1) Literature data used in preparation of Figure 2 and data from ANOVA; (Table S.2) relationship between quantity and type of dispersant and quantity and type of GFM (GNP and GO) incorporated into cementitious composites with the best mechanical performance (PDF)
