# Supplementary material for: Highlights on the Effects of Graphene-Family Materials Dispersion on Hydration and Mechanical Properties of Cement-Based Materials
Source: ACS Omega. 2025 Nov 12;10(46):55161–84. doi: 10.1021/acsomega.5c07530 (PMC12658623; doi:10.1021/acsomega.5c07530)
Supplement: Supplementary file 1 [file ao5c07530_si_001.pdf]

Highlights on the effects of graphene-family materials dispersion on hydration and mechanical properties of cement-based materials

Isis Nayra Rolemberg Prudente[1,3*], Hericles Campos dos Santos[1,4], Jander Lopes Fonseca[2], Jéssica Fernanda Ribeiro Oliveira[1], André Balbino Shibutani[5], Iara de Fátima Gimenez[1], Euler Araujo dos Santos[1,2], Ledjane Silva Barreto[1,2]

[1] *Graduate Program in Science and Engineering Materials, Federal University of Sergipe, São Cristóvão-SE, Brazil*

[2] *Department of Sciences and Materials Engineering, Federal University of Sergipe, São Cristóvão-SE, Brazil*

[3] *Graduate Program in Chemical Engineering, Federal University of Sergipe, São Cristóvão-SE, Brazil*

[4]*Graduate Program in Civil Engineering, Federal University of Sergipe, São Cristóvão-SE, Brazil*

[5] *Graduate Program in Electrical Engineering, Centro Universitário FEI, São Bernardo do Campo-SP, Brazil*

[*] *Corresponding author: Avenida Marcelo Déda Chagas, s/n - Rosa Elze, São Cristóvão-SE, Brazil, 49107-230.*
isis.prudente@outlook.com

Supporting Information

Table S.1 compiles literature data used in the preparation of Figure 2 and data from ANOVA. The dispersant type, the G/D ratio, the UV-vis and Visual Analysis were collected from the reference in the same table.

Dispersant type		G/D	UV-vis (Abs)	Visual Analysis	Ref
MO and PCE		1:0	0.405	-	[1]
		1:0.5	0.400	-	
		1:1	0.440	-	
		1:1.5	0.425	-	
		1:2	0.404	-	
		1:2.5	0.385	-	
PCE		1:0	0.900	-	[2]
		1:0.5	0.920	-	
		1:1	1.015	-	
		1:2	0.960	-	
		1:4	0.905	-	
APEO		1:1	1.016	A	
TNWDIS			1.065	A	
PCE			0.834	D	
PCE			0.556	A	
PCE			0.943	D	
PCE			1.002	D	
GOS	GO	-	-	A	[3]
	GOS	-	-	D	
		1:0	-		[4]
SL		1:5	-	A	
		1:10	-	A	
		1.20	-	A	
PNS		1:5	-	A	
		1:10	-	A	
		1.20	-	A	
PCE		1:5	-	A	
		1:10	-	D	
		1.20	-	D	
PCE		1:0	2.48		[5]
		1:22	2.53		
		1:55	2.56		
		1:77	2.6		
		1:99	2.63		
		1:132	2.63		
		1:176	2.68		
PCE		1:0	-	A	[6]
		1:1	0.3	A	
		1:3	2.5	D	
		1:5	2.3	A	

PCE		1:1	2.75	A	
		1:3	3.2	D	
		1:5	2.6	A	
PCE		1:1	1.8	A	
		1:3	3.6	D	
		1:5	3.3	D	
PCE		1:1	1.4	A	
		1:3	3.0	D	
		1:5	2.8	D	
SGO	GO	-	0.23	A	[7]
	SGO	-	0.9	D	
EVA		1:0	0.18	A	[8]
		1:100	0.52	D	

Obs.: A: Agglomerated D: Dispersed, G/D = GFMs/Dispersion ratio; MO= Methyl orange; GOS = GO functionalized with nanosilica; SL = sodium lignosulfonate; PNS = polycondensate of b-naphthalene sulfonate; SGO = GO functionalized with silane; EVA = Ethylene-vinyl acetate.
Obs_2.: This analysis was performed only with GO

Table S.2. Relationship between the quantity and type of dispersant and the quantity and type of GFM (GNP and GO) incorporated into cementitious composites with the best mechanical performance.

GFM type	Dispersant	Amount of dispersant for the best result (%)	Amount of GFM for the best result (% wt)	Increase in compressive strength (%)	Ref.
**GNP**	MS	0.48	0.06	11.20	[9]
**GNP**	No dispersant	0	0.03	33	[10]
**GNP**	No dispersant	0	0.25	19.82	[11]
**GNP**	No dispersant	0	0.25	35.29	[12]
**GNP**	NS	0.60	0.06	5.0	[9]
**GNP**	NS	1.25	2.50	7.69	[13]
**GNP**	NS	0.61	1.00	-0.2	[13]
**GNP**	PCE	0.24	0.06	8.00	[9]
**GNP**	PCE	1.25	2.50	0.20	[14]
**GNP**	PCE	1.658	0.25	43.3	[15]
**GNP**	PCE	1.50	0.05	13.19	[16]
**GNP**	PCE	0.75	0.08	2.76	[17]
**GNP**	PF127	0.10	0.05	36.27	[18]
**GNP**	SDBS	0.50	0.06	11.00	[19]
**GNP**	SDBS	0.20	0.10	32.51	[18]
**GO**	No dispersant	0	0.02	29.0	[20]
**GO**	No dispersant	0	0.04	14.0	[21]
**GO**	No dispersant	0	0.02	8.90	[3]
**GO**	No dispersant	0	0.03	13.6	[22]
**GO**	No dispersant	0	0.03	46.0	[23]
**GO**	No dispersant	0	0.05	9.71	[24]
**GO**	No dispersant	0	0.05	6.80	[25]
**GO**	No dispersant	0	0.30	32.13	[26]
**GO**	NS	0.02	0.02	30.9	[3]
**GO**	PCE	0.13	0.10	21.0	[27]
**GO**	PCE	0.20	0.06	38.2	[28]
**GO**	PCE	0.60	0.05	9.71	[24]
**GO**	PCE	0.28	0.20	11.2	[29]
**GO**	NS	0.30	0.03	16.1	[30]
**GO**	PCE	0.04	0.02	10.2	[31]
**GO**	PCE	0.058	0.05	47.9	[32]
**GO**	PCE	0.31	0.06	20.62	[33]
**GO**	PCE	0.275	0.10	77.6	[34]
**GO**	PCE	0.20	0.20	16.9	[35]
**GO**	PCE	0.04	0.04	3.13	[2]
**GO**	PCE	0.50	0.06	16.76	[36]
**GO**	PCE	0.75	0.10	4.3	[37]
**GO**	PCE	0.80	0.15	47.7	[38]
**GO**	PCE	3.14	0.075	16.83	[39]
**GO**	PCE	2.00	0.08	34.1	[40]
**GO**	PCE	1.64	0.08	12.65	[41]
**GO**	PCE	0.30	0.03	39.13	[1]
**GO**	PCE	0.04	0.04	6.0	[29]
**GO**	PCE	0.04	0.04	47.62	[4]

* PCE = Polycarboxylate-based superplasticizer, SDBS = Sodium dodecyl benzenesulfonate, PF127 = Pluronic F127, NS = Naphthalene superplasticizer, MS = Melamine superplasticizer.

Reference

[1] Sheng K, Li D, Yuan X. Methyl orange assisted dispersion of graphene oxide in the alkaline environment for improving mechanical properties and fluidity of ordinary portland cement composites. Journal of Building Engineering 2021;43:103166. https://doi.org/10.1016/j.jobe.2021.103166.

[2] Yan X, Zheng D, Yang H, Cui H, Monasterio M, Lo Y. Study of optimizing graphene oxide dispersion and properties of the resulting cement mortars. Constr Build Mater 2020;257:119477. https://doi.org/10.1016/j.conbuildmat.2020.119477.

[3] Lin J, Shamsaei E, Basquiroto de Souza F, Sagoe-Crentsil K, Duan WH. Dispersion of graphene oxide–silica nanohybrids in alkaline environment for improving ordinary Portland cement composites. Cem Concr Compos 2020;106. https://doi.org/10.1016/j.cemconcomp.2019.103488.

[4] Zhao L, Guo X, Liu Y, Ge C, Chen Z, Guo L, et al. Investigation of dispersion behavior of GO modified by different water reducing agents in cement pore solution. Carbon N Y 2018;127:255–69. https://doi.org/10.1016/j.carbon.2017.11.016.

[5] Papanikolaou I, Ribeiro de Souza L, Litina C, Al-Tabbaa A. Investigation of the dispersion of multi-layer graphene nanoplatelets in cement composites using different superplasticiser treatments. Constr Build Mater 2021;293:123543. https://doi.org/10.1016/J.CONBUILDMAT.2021.123543.

[6] Qin W, Guodong Q, Dafu Z, Yue W, Haiyu Z. Influence of the molecular structure of a polycarboxylate superplasticiser on the dispersion of graphene oxide in cement pore solutions and cement-based composites. Constr Build Mater 2021;272:121969. https://doi.org/10.1016/j.conbuildmat.2020.121969.

[7] Nguyen HD, Zhang Q, Lin J, Sagoe-Crentsil K, Duan W. Dispersion of silane-functionalized GO and its reinforcing effects in cement composites. Journal of Building Engineering 2021;43:103228. https://doi.org/10.1016/J.JOBE.2021.103228.

[8] Naseem Z, Shamsaei E, Sagoe-Crentsil K, Duan W. Rheological enhancement of fresh polymer-modified cement composites via surface-modified graphene oxide. Cem Concr Compos 2024;147:105413. https://doi.org/10.1016/j.cemconcomp.2023.105413.

[9] Wang B, Pang B. Mechanical property and toughening mechanism of water reducing agents modified graphene nanoplatelets reinforced cement composites. Constr Build Mater 2019;226:699–711. https://doi.org/10.1016/j.conbuildmat.2019.07.229.

[10] Ababneh AN, Matalkah F, Al-Akhras M. The use of graphene nanoplatelets for enhancement of the compressive strength of mortar containing high-levels of natural Pozzolan. Constr Build Mater 2024;449. https://doi.org/10.1016/j.conbuildmat.2024.138302.

[11] Jaramillo LJ, Kalfat R. Fresh and hardened performance of concrete enhanced with graphene nanoplatelets (GNPs). Journal of Building Engineering 2023;75. https://doi.org/10.1016/j.jobe.2023.106945.

[12] Divya S, Praveenkumar S, Shamir Akthar A, Karthiksoundar N. Performance variation of graphene nanoplatelets reinforced concrete concerning dispersion time. Mater Today Proc 2023. https://doi.org/10.1016/j.matpr.2023.05.104.

[13] Du H, Pang SD. Enhancement of barrier properties of cement mortar with graphene nanoplatelet. Cem Concr Res 2015;76:10–9. https://doi.org/10.1016/j.cemconres.2015.05.007.

[14] Sevim O, Jiang Z, Ozbulut OE. Effects of graphene nanoplatelets type on self-sensing properties of cement mortar composites. Constr Build Mater 2022;359:129488. https://doi.org/10.1016/J.CONBUILDMAT.2022.129488.

[15] Adhikary SK, Rudžionis Ž, Tučkutė S. Characterization of aerogel and EGA-based lightweight cementitious composites incorporating different thickness of graphene platelets. Journal of Building Engineering 2022;57. https://doi.org/10.1016/j.jobe.2022.104870.

[16] Ghani MU, Sun B, Houda M, Zeng S, khan MB, ElDin HMS, et al. Mechanical and environmental evaluation of PET plastic-graphene nano platelets concrete mixes for sustainable construction. Results in Engineering 2024;21. https://doi.org/10.1016/j.rineng.2024.101825.

[17] Park YJ, Lee HS, Seo TS. Experimental Study on Properties of Graphene and Hollow Glass Powder-Added Ultra-High Strength Concrete. Int J Concr Struct Mater 2024;18. https://doi.org/10.1186/s40069-024-00668-x.

[18] Jiang Z, Ozbulut OE, Arce G, Colosi LM. Accounting for decarbonization impacts across the full life cycle of alternative concrete materials: A case-study for graphene-amended cementitious composites. J Clean Prod 2024;482. https://doi.org/10.1016/j.jclepro.2024.144186.

[19] Baomin W, Shuang D. Effect and mechanism of graphene nanoplatelets on hydration reaction, mechanical properties and microstructure of cement composites. Constr Build Mater 2019;228. https://doi.org/10.1016/j.conbuildmat.2019.116720.

[20] Xu G, Du S, He J, Shi X. The role of admixed graphene oxide in a cement hydration system. Carbon N Y 2019;148:141–50. https://doi.org/10.1016/j.carbon.2019.03.072.

[21] Li X, Ming Y, Gui W, Yang C, Sanjayan JG, Hui W, et al. Effects of graphene oxide agglomerates on workability , hydration , microstructure and compressive strength of cement paste. Constr Build Mater 2017;145:402–10. https://doi.org/10.1016/j.conbuildmat.2017.04.058.

[22] Hu M, Guo J, Fan J, li P, Chen D. Dispersion of triethanolamine-functionalized graphene oxide (TEA-GO) in pore solution and its influence on hydration, mechanical behavior of cement composite. Constr Build Mater 2019;216:128–36. https://doi.org/10.1016/j.conbuildmat.2019.04.180.

[23] Gong K, Pan Z, Korayem AH, Qiu L, Li D, Collins F, et al. Reinforcing Effects of Graphene Oxide on Portland Cement Paste. Journal of Materials in Civil Engineering 2015;27. https://doi.org/10.1061/(ASCE)MT.1943-5533.0001125.

[24] Xiong G, Ren Y, Wang C, Zhang Z, Zhou S, Kuang C, et al. Effect of power ultrasound assisted mixing on graphene oxide in cement paste: Dispersion, microstructure and mechanical properties. Journal of Building Engineering 2023:106321. https://doi.org/10.1016/j.jobe.2023.106321.

[25] Jyothimol P, Hazeena R, Issac MT, Mathiazhagan A. Effect of reduced graphene oxide on the mechanical properties of concrete. IOP Conf Ser Earth Environ Sci, vol. 491, Institute of Physics Publishing; 2020. https://doi.org/10.1088/1755-1315/491/1/012038.

[26] Sujitha VS, Ramesh B, Xavier JR. Investigation of functionalized graphene oxide incorporated superabsorbent polymers for enhanced durability, hydration, microstructure and mechanical strength of modified concrete. Compos Interfaces 2024. https://doi.org/10.1080/09276440.2024.2407249.

[27] Long WJ, Wei JJ, Xing F, Khayat KH. Enhanced dynamic mechanical properties of cement paste modified with graphene oxide nanosheets and its reinforcing mechanism. Cem Concr Compos 2018;93:127–39. https://doi.org/10.1016/j.cemconcomp.2018.07.001.

[28] Lv S, Liu J, Sun T, Ma Y, Zhou Q. Effect of GO nanosheets on shapes of cement hydration crystals and their formation process. Constr Build Mater 2014;64:231–9. https://doi.org/10.1016/j.conbuildmat.2014.04.061.

[29] Yang H, Monasterio M, Cui H, Han N. Experimental study of the effects of graphene oxide on microstructure and properties of cement paste composite. Compos Part A Appl Sci Manuf 2017;102:263–72. https://doi.org/10.1016/j.compositesa.2017.07.022.

[30] Liu H, Yu Y, Liu H, Jin J, Liu S. Hybrid effects of nano-silica and graphene oxide on mechanical properties and hydration products of oil well cement. Constr Build Mater 2018;191:311–9. https://doi.org/10.1016/j.conbuildmat.2018.10.029.

[31] Li Z, Xu J, Cao W, Zhang J, Lu D, Yao X. Hydration performance of OWC pastes incorporating GO with various oxygen contents and sizes. Fullerenes Nanotubes and Carbon Nanostructures 2024. https://doi.org/10.1080/1536383X.2024.2390559.

[32] Lv S, Ma Y, Qiu C, Sun T, Liu J, Zhou Q. Effect of graphene oxide nanosheets of microstructure and mechanical properties of cement composites. Constr Build Mater 2013;49:121–7. https://doi.org/10.1016/j.conbuildmat.2013.08.022.

[33] Zhao W, Chen Y, Liu Z, Wang L, Li X. Effects of surface-modified coal-bearing metakaolin and graphene oxide on the properties of cement mortar. Constr Build Mater 2023;372. https://doi.org/10.1016/j.conbuildmat.2023.130796.

[34] Gholampour A, Kiamahalleh MV, Tran DNH, Ozbakkaloglu T, Losic D. Revealing the dependence of the physiochemical and mechanical properties of cement composites on graphene oxide concentration. RSC Adv 2017;7:55148–56. https://doi.org/10.1039/c7ra10066c.

[35] Liu B, Wang L, Pan G, Li D. Dispersion of graphene oxide modified polycarboxylate superplasticizer in cement alkali solution for improving cement composites. Journal of Building Engineering 2022;57. https://doi.org/10.1016/j.jobe.2022.104860.

[36] Kudžma A, Škamat J, Stonys R, Krasnikovs A, Kuznetsov D, Girskas G, et al. Study on the effect of graphene oxide with low oxygen content on Portland cement based composites. Materials 2019;12. https://doi.org/10.3390/MA12050802.

[37] Im S, Cho S, Liu J, Li P, Jin D, Sim S, et al. Comparative effects of graphene oxide on the interfacial transition zone in silica fume-modified cement mortar at varying water-to-binder ratios. Case Studies in Construction Materials 2025;22. https://doi.org/10.1016/j.cscm.2025.e04230.

[38] Reddy PVRK, Prasad DR. The role of graphene oxide in the strength and vibration characteristics of standard and high-grade cement concrete. Journal of Building Engineering 2023;63. https://doi.org/10.1016/j.jobe.2022.105481.

[39] Chu H, Zhang Y, Wang F, Feng T, Wang L, Wang D. Effect of graphene oxide on mechanical properties and durability of ultra-high-performance concrete prepared from recycled sand. Nanomaterials 2020;10:1–17. https://doi.org/10.3390/nano10091718.

[40] Wu YY, Que L, Cui Z, Lambert P. Physical properties of concrete containing graphene oxide nanosheets. Materials 2019;12. https://doi.org/10.3390/MA12101707.

[41] Chen Z, Xu Y, Hua J, Wang X, Huang L, Zhou X. Mechanical properties and shrinkage behavior of concrete-containing graphene-oxide nanosheets. Materials 2020;13. https://doi.org/10.3390/ma13030590.
